# Patient Preferences for Multiple Myeloma Treatments: A Multinational Qualitative Study

**DOI:** 10.3389/fmed.2021.686165

**Published:** 2021-07-06

**Authors:** Rosanne Janssens, Tamika Lang, Ana Vallejo, Jayne Galinsky, Ananda Plate, Kate Morgan, Elena Cabezudo, Raija Silvennoinen, Daniel Coriu, Sorina Badelita, Ruxandra Irimia, Minna Anttonen, Riikka-Leena Manninen, Elise Schoefs, Martina Vandebroek, Anneleen Vanhellemont, Michel Delforge, Hilde Stevens, Steven Simoens, Isabelle Huys

**Affiliations:** ^1^Department of Pharmaceutical and Pharmacological Sciences, KU Leuven, Leuven, Belgium; ^2^Myeloma Patients Europe, Brussels, Belgium; ^3^Department of Haematology, H. Moises Broggi/ICO-Hospitalet, Barcelona, Spain; ^4^Department of Hematology, Helsinki University Hospital Comprehensive Cancer Center, Helsinki, Finland; ^5^University of Helsinki, Helsinki, Finland; ^6^Carol Davila University of Medicine and Pharmacy, Bucharest, Romania; ^7^Fundeni Clinical Institute, Bucharest, Romania; ^8^Association of Cancer Patients in Finland, Helsinki, Finland; ^9^Faculty of Economics and Business, KU Leuven, Leuven, Belgium; ^10^University Hospital Leuven, Leuven, Belgium; ^11^Institute for Interdisciplinary Innovation in Healthcare (I3h), Université Libre de Bruxelles (ULB), Brussels, Belgium

**Keywords:** multiple myeloma, patient preferences, nominal group technique, qualitative research, attributes, drug development, regulatory benefit-risk assessment, health technology assessment

## Abstract

**Background:** Investigational and marketed drugs for the treatment of multiple myeloma (MM) are associated with a range of characteristics and uncertainties regarding long term side-effects and efficacy. This raises questions about what matters most to patients living with this disease. This study aimed to understand which characteristics MM patients find most important, and hence should be included as attributes and levels in a subsequent quantitative preference survey among MM patients.

**Methods:** This qualitative study involved: (i) a scoping literature review, (ii) discussions with MM patients (*n* = 24) in Belgium, Finland, Romania, and Spain using *Nominal Group Technique*, (iii) a qualitative thematic analysis including multi-stakeholder discussions.

**Results:** MM patients voiced significant expectations and hopes that treatments would extend their lives and reduce their cancer signs and symptoms. Participants however raised concerns about life-threatening side-effects that could cause permanent organ damage. Bone fractures and debilitating neuropathic effects (such as chronic tingling sensations) were highlighted as major issues reducing patients' independence and mobility. Patients discussed the negative impact of the following symptoms and side-effects on their daily activities: thinking problems, increased susceptibility to infections, reduced energy, pain, emotional problems, and vision problems. MM patients were concerned with uncertainties regarding the durability of positive treatment outcomes, and the cause, severity, and duration of their symptoms and side-effects. Patients feared short-term positive treatment responses complicated by permanent, severe side-effects and symptoms.

**Conclusions:** This study gained an in-depth understanding of the treatment and disease-related characteristics and types of attribute levels (severity, duration) that are most important to MM patients. Results from this study argue in favor of MM drug development and individual treatment decision-making that focuses not only on extending patients' lives but also on addressing those symptoms and side-effects that significantly impact MM patients' quality of life. This study underscores a need for transparent communication toward MM patients about MM treatment outcomes and uncertainties regarding their long-term efficacy and safety. Finally, this study may help drug developers and decision-makers understand which treatment outcomes and uncertainties are most important to MM patients and therefore should be incorporated in MM drug development, evaluation, and clinical practice.

## Introduction

Patient preference studies use qualitative and quantitative methods to understand which treatment and disease-related characteristics (efficacy outcomes, side-effects, and symptoms) are important to patients, how important they are to patients, the trade-offs patients are willing to make between these characteristics, and how preferences may vary according to individual patient characteristics (1–3). Stakeholders involved in drug development and evaluation—such as drug developers, regulators, *Health Technology Assessment* (HTA) bodies and payers—have acknowledged the potential value of using patient preference studies to inform their respective decisions (4–6). More specifically, patient preference studies could: (i) reveal the patient perspective on unmet treatment needs in early drug development, (ii) inform the development of patient reported outcome measures and the selection of clinical trial endpoints, (iii) help understand which are the key favorable and unfavorable effects and uncertainties in regulatory benefit-risk assessment, (iv) quantify the relative importance of treatment characteristics in HTA and payer decisions, and (v) inform the development of decision aids used in shared individual treatment decision-making between patients and clinicians (1, 4–18).

On the regulatory level, the *European Medicines Agency* (EMA) and the *US Food and Drug Administration* (FDA) intend to systematically include preference studies in regulatory benefit-risk assessment (4–6, 19–21). Mirroring the FDA's efforts toward guidance surrounding patient preference studies, the EMA aims to develop guidance on appropriate methods for patient preference study design, conduct, analysis, and presentation for regulatory purposes, to ensure that high quality methodologies are applied (4). A reflection paper by the EMA details opportunities for the development of new guidelines by the *International Council for Harmonization of Technical Requirements for Pharmaceuticals for Human Use* (ICH). These guidelines will aim to provide a globally harmonized approach to inclusion of the patient's perspective in a way that is methodologically sound and sustainable for both regulated industry and regulatory authorities (22). From the HTA/reimbursement perspective, the *National Institute for Health and Care Excellence* (NICE) argues that patient preference studies could be used to inform the selection of clinical trial endpoints, and inform regulatory benefit-risk assessment, echoing the EMA's viewpoints (23). In addition, NICE sees a role in preference studies for informing their HTA assessment alongside other types of clinical and economic evidence (23, 24). Similarly, drug developers more often include preference studies in their drug development plans, regulatory, and HTA submissions (25).

However, while stakeholders have expressed an interest in using patient preference studies to inform their respective decisions, previous research has revealed that more evidence-based preference study development is needed to build methodological and practical knowledge and address uncertainties regarding the design, conduct, and use of patient preference studies (1, 10, 26). In response to this, several research projects, such as the IMI PREFER project, have been initiated by drug developers, academic researchers, as well as HTA bodies and regulatory agencies. Such projects aim to investigate how patient preference studies could inform decision-making, and how such studies could be designed to meet methodological requirements of stakeholders involved in these decisions (27–29).

A crucial initial step in patient preference studies is the use of qualitative data collection for identification of the key attributes and levels of importance to patients for inclusion in the subsequent quantitative phase of the study. Attributes are the key aspects that impact patients' choices toward treatments and include benefits, risks or other clinical and non-clinical aspects that influence the desirability or acceptability of medical interventions (30). Therefore, attributes of key importance to patients may align decision-making with patient's perspectives both in the individual treatment decision-making context (16–18), as well as in decision-making regarding drug development, authorization, and reimbursement. Attribute levels are the values or categories used to characterize the performance of a treatment (31). As qualitative methods provide in-depth and meaningful information from patients, their use is recommended for the development of attributes and levels. Qualitative methods with patients may reduce the potential for misspecification of attributes through overreliance on the views of experts and researchers (27, 28). In doing so, using qualitative research for the development of attributes and levels may improve the validity of subsequent quantitative preference surveys. Therefore, by combining both qualitative and quantitative methods in preference studies, the data collected on patient preferences is likely to be more comprehensive, meaningful, and a valid interpretation of the true patient perspective (32). However, qualitative preference research that informs subsequent preference surveys remains underreported, creating uncertainty regarding the methodological and practical application of these methods and results for informing subsequent quantitative preference surveys.

Eliciting preferences from *Multiple Myeloma* (MM) patients is especially valuable in view of the rapid development of various novel MM treatments with substantial effects on survival, toxicity, efficacy, and related long-term uncertainties. Among patients and various stakeholders, the impact of these treatments on patients' lives, attitudes and choices toward treatments is largely unknown. MM is the second most frequent hematological malignancy after non-Hodgkin lymphoma, accounting for 1% of all cancers and 10% of blood cancers (33, 34). MM is characterized by a proliferation of plasma cells in the bone marrow, typically accompanied by the secretion of monoclonal immunoglobulins (M-proteins or paraproteins) (35). This proliferation causes symptoms such as skeletal damage, hypercalcemia, renal insufficiency, anemia, and infections (36). Because MM disrupts the normal functioning of the bone marrow, damages the bones and causes kidney failure, MM is considered to be a debilitating and life-threatening disease. Despite several drugs being available, MM has been labeled an incurable disease and only half of the diagnosed patients live longer than 5 years (33).

New MM treatments are currently being developed that have different side-effect profiles, mechanism of action, and efficacy from those currently available. More specifically, innovative treatments currently under development, such as bispecific T-cell engagers and chimeric antigen receptor therapies (CAR-T), have shown to be efficacious but also associated with severe risks such as cytokine release syndrome (an acute systemic inflammatory syndrome characterized by fever and multiple organ dysfunction) and neurotoxicity's (such as encephalopathy, aphasia, delirium, tremor, and seizures) (37). Differences between treatments, based on varying benefits and risks, raise the question about how MM patients value these treatment aspects. Furthermore, decisions surrounding MM treatment can be labeled as “preference sensitive” decisions where: (i) multiple treatment options exist and there is no option that is clearly superior for all patients; (ii) the evidence supporting one option over others is considerably uncertain or variable and (iii) patients' views about the most important benefits and acceptable risks of a treatment vary considerably within a population and may differ from those of healthcare professionals (20).

Therefore, given the lack of valid, meaningful, and comprehensive qualitative research on MM patient preferences, the present study aimed to understand which characteristics MM patients find most important, and hence should be included as attributes and attribute levels in a subsequent preference survey. By pursuing these objectives, this study derives experience-based learnings regarding the design, conduct and analysis of qualitative research aiming to develop attributes and levels for inclusion in subsequent preference surveys in the context of drug development and evaluation. Such methodological learnings may foster the development of a standardized approach to be used by all stakeholders across disease areas, and serve to include a validated patient preference framework for drug development, allowing for future comparisons of patient preference studies and their results.

## Materials and Methods

### Study Context

The *Patient Preferences in Benefit-Risk Assessments during the Drug Life Cycle* (PREFER) project is a 6-year public private partnership that received funding from the *Innovative Medicines Initiative* (IMI) 2. This project seeks to guide drug developers, regulatory authorities, and HTA bodies (including reimbursement agencies and payers) on how and when patient preference studies should be performed and how the results can be used to inform decision-making. The initial phase of the PREFER project included discussions with a broad representation of stakeholders such as patients, patient organizations, regulatory authorities, HTA bodies, and reimbursement agencies that expressed interest in preference studies, and revealed the need to further explore and test preference methods (1, 7, 9, 10, 26). Therefore, this study has been developed in the context of recommendations formulated by IMI PREFER. The results of PREFER are expected to lead to changed practices, in that stakeholders will routinely assess whether a preference study would add value at key decision points in the medicinal product life cycle and, if so, implement patient preference studies according to the PREFER project recommendations (38).

### Study Design

This study was designed and executed according to: (i) the recommendations on qualitative data collection and analysis methods for initial attribute development (39, 40); (ii) the steps describing attribute and level development in health preference research formulated by Bridges et al. (41); (iii) the criteria described by Hensher et al. (42) regarding what constitutes “good” attributes; (iv) the framework method for thematic analysis described by Lacey and Luff (43) and (v) the recommendations for reporting the results of a qualitative preference study (40).

Following recommendations by Hollin et al. (40), Coast et al. (39), and Bridges et al. (41), this paper describes: (i) the rationale for the methodological steps and choices taken to develop attributes and levels; (ii) a detailed description of the included participants; (iii) details regarding the practical steps and setting of the qualitative study including the recruitment, discussion guides, involved researchers; (iv) details of the subsequent steps including the transcription, translation, and analysis and (v) the results of the qualitative study.

### Study Phases

**Figure 1:**
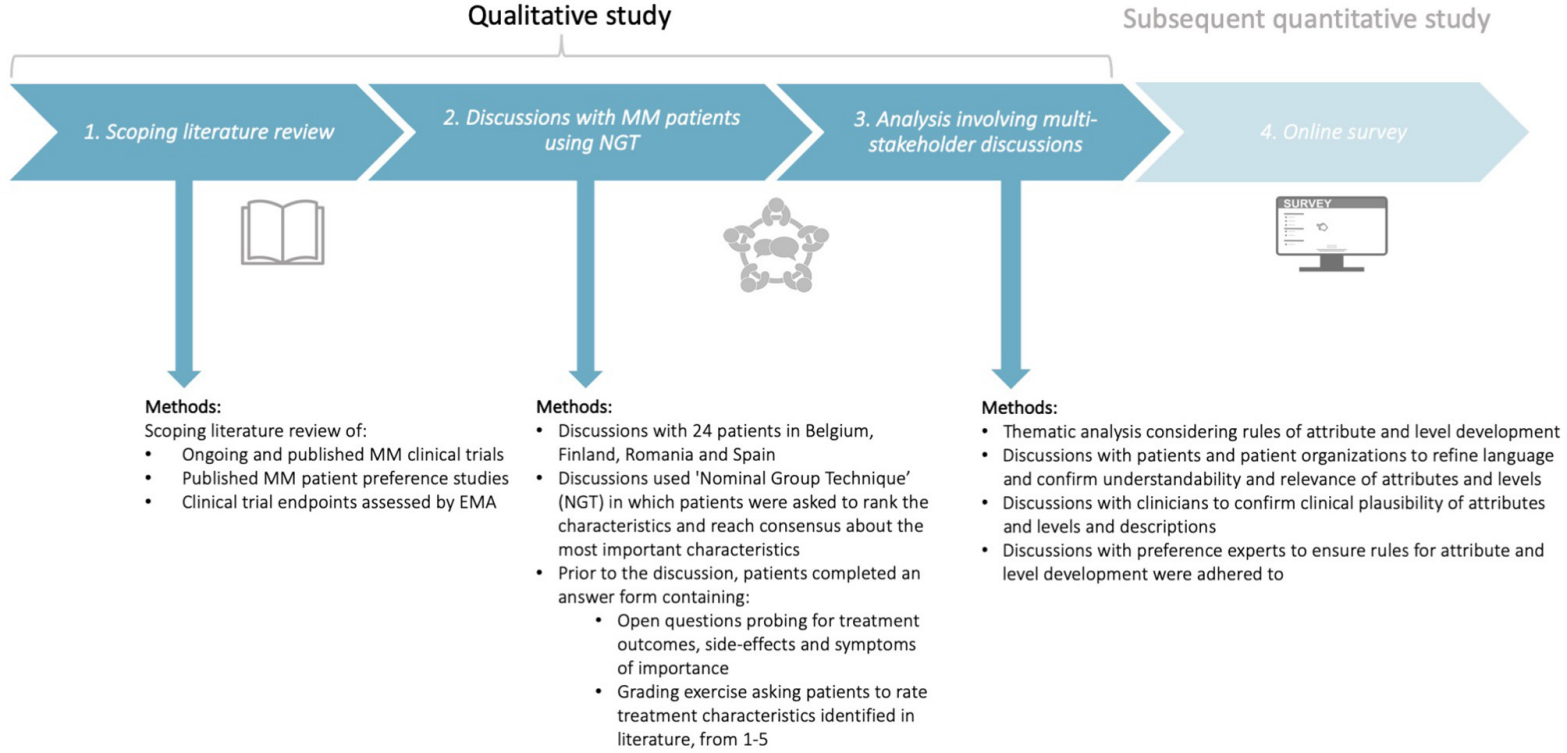
Design of the qualitative study consisting of three phases: (i) a scoping literature review, (ii) discussions with MM patients using Nominal Group Technique (NGT), and (iii) analysis involving multi-stakeholder discussions with patients, patient organizations, clinicians, and preference experts. The subsequent quantitative study will be conducted using the attributes and levels identified in the present qualitative study. MM, Multiple Myeloma; NGT, Nominal Group Technique; EMA, European Medicines Agency.

To understand the key characteristics of importance to MM patients, a qualitative study was completed in three phases (Figure 1). Several preference studies attest to the usefulness of qualitative methods with patients and advocate for the use of literature reviews to inform the development of attributes and levels (31, 39–41, 44). Therefore, this study involved three phases, whereby each phase informed the subsequent phase (Figure 1): (i) a scoping literature review, (ii) discussions with MM patients using *Nominal Group Technique* (NGT), and (iii) a combined quantitative and qualitative thematic analysis involving multi-stakeholder discussions with patients, patient organizations, clinicians, and preference research experts.

#### Phase 1: Scoping Literature Review

The scoping review aimed to identify potential relevant characteristics (treatment outcomes, symptoms, and side-effects) for grading in subsequent patient discussions using NGT. Bridges et al. (41) recommend that attribute development should be supported by evidence on the potential range of preferences and values that respondents of the preference survey may hold. Therefore, the list of treatment characteristics that was used for the NGT (Appendix 1, section 3) was informed by a scoping literature review of: (i) the attributes and key results of published preference studies conducted among MM patients (Appendices 2, 3), (ii) favorable and unfavorable effects of MM treatments already assessed by EMA that includes characteristics of treatments already being prescribed to patients (Appendices 4, 5) and (iii) primary and secondary endpoints and adverse events reported in phase 3 MM clinical trials in the European Union (EU) to ensure the attribute list captured treatment characteristics of therapies in development; this was done so that in the discussions patients would be able to discuss potential “future” treatment outcomes and side-effects, even though they had not yet experienced them (Appendices 6, 7).

Searches for published preference studies among MM patients were conducted in PubMed and Embase (see Appendix 2 for the selection procedure). The search queries included free text terms in title and/or abstract and *Medical Subject Headings* (MeSH) and included synonyms for the following two concepts: “multiple myeloma” AND “patient preferences.” The database searches yielded 250 publications. Publications were included if they reported preferences from MM patients. Conversely, studies were excluded if: (i) preferences were not elicited from patients (e.g., only from caregivers or clinicians); (ii) no preference method (qualitative/quantitative) was applied; (iii) no preferences were reported (e.g., study protocols) and (iv) the study focused on: patient preferences for whether or not patients are willing to participate in decision-making, patient preferences for remote monitoring, or if the study investigated whether patients do or do not want to receive information. The results were screened in a 2-fold manner. First, the title and abstract were screened based on the in- and exclusion criteria. Afterwards, the full text was reviewed against the same criteria. From the database searches, 15 publications were included in the review. Subsequently, the following information was extracted (Appendix 2): (i) first author and year; (ii) type of publication; (iii) research objective; (iv) participants; (v) preference method(s) used; (v) attributes/items/factors identified or used in the study and (vi) key results. To develop a final list of characteristics for grading (Appendix 1), the treatment characteristics that emerged from the scoping review were combined and then grouped with both the characteristics of treatments being prescribed to patients (Appendices 4, 5) and with the characteristics of treatments included in phase 3 MM clinical trials (Appendices 6, 7).

#### Phase 2: Discussions With Multiple Myeloma Patients Using Nominal Group Technique

##### Objectives and Rationale

Phase 2 aimed to: (i) understand which characteristics, including those identified in phase 1, were most important to MM patients, and hence should be included as attributes in the subsequent preference survey that quantifies the relative importance of these attributes; (ii) understand the factors and dimensions influencing patient choices; what determines whether patients would take, not take, or discontinue a certain treatment, and hence should be included as attribute levels and (iii) understand the language patients use to describe symptoms, treatment outcomes, and side-effects, and hence should be the language used to describe the attributes and levels.

To reach these objectives, discussions with 24 MM patients in Belgium, Finland, Romania, and Spain were held (see “Recruitment, study population, and setting” for a rationale for including these countries). The discussions used NGT, a type of focus group discussion methodology, that asked patients to rank and reach consensus on the most important characteristics (see “step-by-step procedures”). While standard focus group discussions use open discussion throughout, NGT is a consensus focus group methodology that differs from standard focus group discussions. In addition to providing a format for open discussion, NGT includes a structured four-stage process and a methodology for capturing participant responses and with inclusion of prioritization and participant's individual and collective perspectives. NGT is specifically suited to identify attributes due to its structured approach and grading methodology; the grading allows researchers to select and understand which treatment characteristics are most important and hence should be used for developing the attributes in the subsequent preference survey. Furthermore, NGT has the advantage over other qualitative consensus methods as it ensures groups to reach consensus in a short period of time (39).

##### Patient Involvement and Piloting

In addition to inclusion in NGT discussions, MM patients, and MM patient organizations were involved in all steps of research. MM patients and/or MM patient organization members provided written and oral feedback on all patient materials including the information sheet, informed consent, answer sheet (including explanatory parts), and questions. All patient documents were first translated by a professional translation company to the native language of participants, and subsequently revised for accuracy and understandability by patients, patient organizations, and clinicians.

##### Recruitment, Study Population, and Setting

Hematologists performed the recruitment at their respective hospitals and were asked to ensure a diverse patient population was invited to participate in the discussions. It was anticipated that several individual patient characteristics—such as socio-demographics, disease stage and treatment experience—could influence participants' opinions and rankings. The goal was to ensure that the attributes and levels identified in this study were not directed only to patients with a specific treatment exposure, disease history, age or country of origin; but rather toward all patients along the MM spectrum. Therefore, during recruitment, heterogeneity in terms of treatment experience, disease stage, age, and country was introduced as much as possible. During sampling, hematologists used the following inclusion criteria: (i) patients diagnosed with symptomatic MM; (ii) patients ability to understand the language to be used in the discussion and (iii) patients ability to participate in the discussion.

Recruitment sought to include between 5 and 7 MM patients across four countries: Belgium, Finland, Romania, and Spain. These countries were included to account for potential differences in patient characteristics and, as mentioned above, to increase heterogeneity and thereby ensure the identified attributes and levels were not only relevant to a particular type of patient. While McMillan et al. (45) describes that most NGTs include between 2 and 14 participants, a maximum of seven is recommended as a much larger number would delay the phased process of the NGT discussion, which aims to reach consensus in a short time span (up to 2 h). Therefore, minimally 5 and maximally 7 patients were included in each country. There are no guidelines that define how much data, and hence, participants should be included in qualitative research (30). Instead, saturation is often used to define when data collection can stop (30, 32, 46). Saturation is defined as the point when “*no new information or themes are observed in the data”* (47). Hennink et al. (48) state that when the goal is to identify “core” issues, few discussions could be enough to reach data saturation, and some studies have reached saturation after 4–6 focus groups (30, 32, 46). Since the goal of our study was to identify core, overarching attributes, it was expected that data saturation could be achieved by including between 5 and 7 MM patients in four countries (*n* = 24 across all countries). Following qualitative data collection, it appeared that the same themes of treatment attributes were observed across different countries. Hence, it was decided that saturation was reached and no additional data was needed to inform the attributes and levels.

The discussions were organized at a location convenient for participants, between April and November 2020, and considered the implications of the coronavirus (COVID-19) pandemic; discussions were organized either face-to-face or online, according to the preference of participants and recommendations set-out by hospitals regarding patient contact.

##### Step-by-Step Procedures

As part of the recruitment process, an invitation letter was sent to those expressing interest in the study and fulfilling inclusion criteria (see “Study recruitment, population and setting”). Potential participants were contacted to verify their willingness to participate and if so, arrange the practicalities of the discussion. The information sheet and informed consent was provided to participants in their own language. Both documents were provided to participants prior to the discussion, and the informed consent form was signed by all participants before the discussion. As preparation for the discussion, participants were invited to complete an answer sheet containing three sections: (i) participants' background characteristics, including Chew's Set of Brief Screening Questions (Appendix 1, section 1); (ii) open questions probing for treatment characteristics of importance (Appendix 1, section 2) and (iii) a grading exercise asking patients to grade the treatment characteristics identified in the scoping literature review from 1 (= not important at all) to 5 (= very important) (Appendix 1, section 3). The NGT discussion consisted of the following four steps (Appendix 8): (i) idea, (ii) round robin, (iii) clarification and finalization of the list of attributes, (iv) grading and consensus.

Each discussion was conducted by a person fluent in the native language of the participants. The discussions lasted around 90 min, were voice-recorded and included a break of ~10 min. The audio-recordings were transcribed verbatim by a professional transcribing company in the original language and then translated to English. To ensure patients' opinions were accurately reflected in the subsequent analysis and development of the attributes and levels, the moderators were closely involved in the subsequent analysis (see phase 3).

#### Phase 3: Analysis Involving Multi-Stakeholder Discussions

In the final phase, a combined quantitative descriptive analysis of patients' rankings and iterative qualitative thematic analysis of the discussion transcripts was used to determine overarching themes of prioritized treatment characteristics relevant to all participating patients, regardless of their treatment exposure, disease history, age, or country of origin.

##### Quantitative Analysis

Participants' self-reported characteristics, as obtained through sections 1 and 3 of the answer sheet (Appendix 1) were analyzed descriptively using Microsoft Excel. Patient characteristics were tabulated for all patients together and for each of the questions asked in Appendix 1. ANOVA and Fischer exact tests were performed to investigate statistically significant differences between groups of participants and countries. Health literacy was determined using Chews' set of brief screening questions (49). The grades for the characteristics were calculated per country to derive rank orders and averages at country level. To obtain a final rank of the themes pertaining to treatment characteristics, the averages for each theme were calculated by combining the previously calculated averages obtained in the four countries.

##### Qualitative Analysis

The qualitative analysis took into account the following criteria and best practices for attribute and level development; therefore, attributes and levels should be:

- Relevant to patients and/or policy-makers, plausible, capable of being traded, unambiguous, distinctly different from other included attributes, comprehensive, and of salience to respondent's decisions (39, 40);
- Inclusive of all aspects that might be important for an individual in coming to a decision (28, 39);
- Not too close to the latent construct such as overall quality of life (28);
- Not have such a large impact on decisions that large numbers of respondents of the quantitative survey make no errors when deciding, such as overall happiness with the alternative treatments presented in the preference survey (28);
- Not intrinsic to a person's personality, these aspects need to be considered in analyzing and describing preference heterogeneity (28);
- Developed through an iterative, constant comparative analysis approach to continually modify and extend the attributes and levels to ensure that all key aspects can be incorporated through this modification (28);
- Inclusive of all aspects that might be important for an individual in coming to a decision, as ignoring important attributes and levels may bias findings; and qualitative methods to determine overarching attributes must encompass key themes combined with piloting to avoid bias (28);
- Created through a process consisting of conceptual development where the attributes and levels are identified, followed by refinement of language to ensure the intended meaning is conveyed toward the participants in the preference survey (28);
- Inclusive of all characteristics that potentially characterize the alternative treatments presented to participants in the preference survey, with consideration that some characteristics may be excluded if the alternative treatments are not plausible to subjects (39).

The framework method by Lacey and Luff (43) was used to develop overarching themes that capture prioritized characteristics for inclusion as attributes and levels (Table 1). The analysis was performed by a multi-stakeholder team including patients, patient organizations, clinicians, and academic preference research experts. Discussions with patients and patient organizations specifically sought to confirm whether the themes captured the most relevant characteristics for inclusion as attributes and levels, and whether the results described accurately represented their views. In particular, MM patients and/or MM patient organization members provided written and oral feedback on the relevance, comprehensiveness, and understandability of the themes of characteristics for inclusion as attributes and levels. Discussions with clinicians were held to confirm the clinical plausibility of the attributes and levels. Also, to ensure adherence to rules for attribute and level development, preference expert input was included. Finally, the attributes and levels were reviewed by MM patients to receive end-user feedback.

**Table 1:** Iterative steps of the qualitative thematic analysis using the framework method.

1. Familiarization	The transcripts were thoroughly read and re-read and the audio-recordings were listened to again if a certain part of the transcript was unclear. The margins of the transcripts were used to write down analytical notes, thoughts or impressions (e.g., when participants expressed exceptionally strong or contrasting views). Discussions among the moderators on preliminary findings sought to confirm whether they interpreted the discussions in the same manner.
2. Identifying a thematic framework	A list of overarching themes capturing prioritized treatment characteristics was developed based on the views expressed by patients during the discussions, in their answer sheet, and the ranked list of characteristics and explanations. This list was transported to NVivo (11th edition, QSR International) for coding.
3. Coding	Literal quotations (text) in the transcript and answer sheets were attached to each of the themes, describing what participants stated about these themes (coding). Throughout the coding process, it was assessed whether the list of themes and their explanations covered what participants said. If not, modifications were made to the name of the theme. Discussions among the team (see below) sought to modify and reach consensus about the themes. The end result was the final list of themes, each with a brief explanatory description of their meaning including examples to further explain the theme (see below).
4. Charting	NVivo was used for charting (summarizing) the data per attribute.
5. Mapping and interpretation	Several meetings among a multi-stakeholder team were organized to discuss the qualitative findings (the final list of themes) together with the characteristic scorings of the quantitative analysis. During these meetings, consensus was reached about the final list of attribute themes and associated levels to take forward to the quantitative survey.

*An iterative, constant comparative analysis approach was used to allow for continuous modifications and extensions of the themes to ensure that all key aspects of importance to patients could be incorporated*.

## Results

### Participants' Characteristics

In total, 24 MM patients (6 per country, 4 countries) agreed to participate. The average response rate across countries was 46%. Reasons for not participating were: (i) research topic was not in their field of interest; and (ii) not willing to communicate in groups. The mean age across countries varied between 60 and 65 years (M: 61 across all countries, range 46–73). Most participants had a Masters' degree (42%) followed by a Bachelor (25%) or High school degree (25%). Most participants (58%) described their activity level as “*not my normal self, but able to be up and about with fairly normal activities” (fair mobility)*, followed by “*normal, without any limitations” (no limitations;* 25%) and 13% identified themselves as “*not feeling up to most of the things, but in bed of chair less than half of the day”* (sedentary) (Appendix 9). Most participants (88%) did not live alone at the time of the discussion. Many participants were employed (50%) or retired (42%). The median participant received their MM diagnosis at the age of 55. Participants were heterogeneous in terms of how long they had received their diagnosis; ranging between 18 years ago to the same year of the discussion.

Nearly all patients (96%) were on treatment at the time of the discussion. Across countries, participants were most frequently treated with proteasome inhibitors (PIs) (50%), immunomodulating agents (IMiDs) (46%) and steroids (46%), followed by supportive treatments (33%, e.g., calcium and vitamin D supplements, pain medication) and monoclonal antibodies (mAbs) (21%). Participants were previously treated with an average of three different regimens (one treatment line referring to one drug or a combination of drugs given for a specific time duration) at the time of the discussion and the number of treatment lines across participants ranged between one and seven previous treatment lines. Fifty-eight percentage of patients indicated that they suffer from at least one of the following chronic health problems: heart rhythm disorders, prostate hypertrophy, hypertension, hypothyroidism, glaucoma, renal insufficiency, diabetes, and arthritis. The vast majority (83%) of participants indicated that they were not in frequent contact with a patient organization. Across countries, the majority of participants had a high (46%) or moderate (42%) health literacy.

Statistical tests (Fischer exact and ANOVA) revealed no significant differences between the patient groups of the different countries, regarding their age (*F* = 1.61, *p* = 0.22), gender (*X*^2^ = 1.90, *p* = 0.81), education (*X*^2^ = 8.01, *p* = 0.62), work status (*X*^2^ = 10.76, *p* = 0.05), contact with patient organizations (*X*^2^ = 3.72, *p* = 0.22), other chronic health problems (*X*^2^ = 6.73, *p* = 0.08), health literacy (*X*^2^ = 2.99, *p* = 0.93), and number of treatment lines (*F* = 2.64, *p* = 0.09).

However, there were statistical differences in activity level (*X*^2^ = 10.78, *p* = 0.03), living situation (*X*^2^ = 4.70, *p* = 0.04), enrolment in clinical trial (*X*^2^ = 8.33, *p* = 0.04), and years since diagnosis (*F* = 3.28, *p* = 0.04). In particular, the Finnish group had a better activity level than the other groups; 67% of the participants considered their activity level as “*normal, without any limitations.”* Further, only 50% of the Finish group was not living alone vs. 100% in the other countries. Regarding clinical trial enrolment, nearly all Finnish participants were currently (50%) or previously (30%) enrolled in a clinical trial, as opposed to the other countries, where nearly equal distributions were observed (42% yes vs. 54% no). Spanish participants were more recently diagnosed with MM (M: 1 year since diagnosis) vs. Belgian participants (M: 9 years) (see Appendix 9 for a full overview of participants' characteristics).

### Themes Capturing Prioritized Characteristics for Inclusion in Attributes and Levels

Patients across countries and with varying disease and treatment experiences reached consensus on the importance of the following eleven attribute themes (outcomes, side-effects, and symptoms): life expectancy, life-threatening side-effects, treatment response, mobility problems, thinking problems, infections, reduced energy, pain, emotional problems, eating and digestive problems, and vision problems. These attribute themes are categorized below into: (i) favorable effects: treatment outcomes considered desirable or beneficial, and (ii) unfavorable effects: side-effects and symptoms negatively impacting patients' life expectancy and/or quality of life.

Regarding the types of attribute levels, patients highlighted the importance of specifying the duration and severity of the treatment effects as it would determine their treatment choices. In particular, patients expressed the fear of short-term positive treatment outcomes and long-term negative effects. Conversely, several expressed that transient side-effect are more acceptable. Patients were also afraid of symptoms and side-effects that are so severe, in that sense that they would hamper them in performing their daily activities. Therefore, severity and duration were specified as attribute levels.

Patients highlighted the importance of both life expectancy and quality of life: “*as many years with the best quality of life as possible”* and highlighted that the balance between the treatments' effect on their life expectancy and quality (would) determine their individual treatment choices: “*I would reconsider continuing treatment) if the impact on daily life is so great that the quantity of life (years) becomes less important than the quality of life.”* Participants' willingness to accept a reduction in quality of life in return for an increased life expectancy differed across participants and depended on their individual situation and personal attitudes. In particular, participants who were older, had undergone more treatments, and had no young children, seemed to place more importance on quality of life related attributes (such as pain) rather than life expectancy, and vice versa. However, on average, life expectancy was graded the highest by MM patients, followed by side-effects and symptoms that significantly impact patients' life expectancy and/or quality of life.

#### Favorable Effects: Treatment Outcomes Considered Desirable or Beneficial

##### Life Expectancy

Increasing life expectancy was on average, the most important treatment outcome for patients across the four countries: “*Lengthened life span is of course most important,” “I think that the most desirable effect of myeloma treatment would be longer life, and, I don't know whether this needs any justification as to why.”* Depending on their personal context, participants described that they want to be there to see their children grow up, take care of their loved ones and be professionally active. MM patients described the negative psychological impact of the uncertainty of how long they had to live: “*the sword of Damocles hanging over me*.”

##### Treatment Response

Participants voiced significant expectations and hopes that treatments would work to successfully fight their cancer and extend their lives. Any improvement or positive treatment outcome was considered to be important: “*Any improvement would be welcome.”* Participants agreed that they want a treatment that will have lasting improvements in any signs and symptoms associated with their cancer and removal of cancer cells.

Many patients hoped for a complete remission, i.e., a complete removal of all cancer signs. Several participants acknowledged that cure—a complete and permanent elimination of all cancer cells—is not achievable with current treatments. However, to be cured permanently, was considered to be the ultimate treatment goal by some patients: “*I am hoping that by continuing the treatment I will get cured*.” Several also described that if the treatment would cure them, they would be willing to accept even those treatment side-effects that they had described as the worst treatment effects.

Positive laboratory and imaging tests were recognized as important indicators of a stabilization of cancer progression. The knowledge and interest of some participants regarding MM biomarkers was remarkable; several participants shared detailed experiences regarding their test results and the importance of positive laboratory and imaging findings. Patients also highlighted that test results impact their psychological well-being; despite burdensome symptoms and side-effects, positive results give patients hope and motivation to carry on: “*To get that M-component to fall or become invisible; these are such improvements that they do make even some more difficult side-effects acceptable.”* The importance of a sustained positive treatment response (inclusive of reduction in symptoms) was also noted.

#### Unfavorable Effects: Side-Effects and Symptoms Negatively Impacting Patients' Life Expectancy and/or Quality of Life

##### Life-Threatening Side-Effects

Patients were afraid of serious side-effects that are life-threatening and could (permanently) damage other (vital) organs: “*Of course, any life-threatening side-effects (…) would make me think twice (…), as the side-effects would then be worse than the illness.”* The following side-effects were raised: developing another cancer, stroke, heart failure, septic shock, and severe bleeding. Among these, the fear and uncertainty of developing another cancer was highlighted multiple times: “*You also have psychological consequences, the fear of a secondary cancer.”*

##### Mobility Problems

Participants discussed which physical symptoms and side-effects significantly reduce their independence and “*control their life*.” In particular, bone fractures were highlighted as major issues reducing patients' ability to move, and hence, reducing their independence and overall quality of life. Regarding bone fractures, several patients expressed the desire for an improvement in their “bone weakness” or a stabilization of their bone destruction. Aside from the negative *physical* impact of bone weakness, patients also described the negative *psychological* impact of these issues; the fear of being active due to a high risk of fractures: “*So it's the fear to try to do something and get a fracture, like that, break a bone, that fear.”* Vice versa, patients argued that an increased ability to move would improve their psychological well-being. Some participants added that bone pain could be both due to the disease as well as due to treatment with bisphosphonates. The importance of the duration and severity of their mobility problems was also stressed; while some patients expressed to be willing to accept temporary immobility, permanent immobility—requiring a rollator or wheelchair—was considered to be unacceptable by many. Patients also expressed the burden of the uncertainty of the duration of these problems: “*When will I be able to ski? Will I ever be able to ski? To do the things we did before.”*

Symptoms associated with nerve damage and subsequent mobility problems were also discussed extensively. These issues commonly occur in extremities [feet, legs (calves), hand, fingers] and are particularly burdensome because they limit patient movement. Examples of these symptoms are: chronic (strange) sensations or tingling (“pins and needles”); over-sensitivity of the skin and bruises; numbness or reduced physical sensations (i.e., “sleeping” feet, fingers, and toes). Furthermore, participants described weakness or stiffness in feet and legs causing instability: “*Not having tingling in the feet. It makes walking difficult for me, the feeling of always having numb feet is very unpleasant.”* Participants argued how improvements in these problems would be highly welcome: “*If something was found that would improve the whole tingling sensation that has become chronic.”* Participants hoped these effects would improve once the treatment is stopped. Several feared constant mobility problems related to neuropathic symptoms and raised the uncertainty related to the duration of these issues. Those participants who themselves did not experience permanent side-effects, admitted to the psychological trauma of watching and knowing other MM patients who were permanently immobile and dependent on others.

##### Thinking Problems

Patients expressed fear of cognitive changes that would affect their daily mental activities such as: difficulties to think clearly, concentrate and pay attention (e.g., difficulties in reading a book, watching TV), memory loss, lower levels of consciousness, hallucinations (seeing, feeling, or sensing things that seem real but are not), dizziness, and confusion. Patients feared permanent and severe thinking problems that may reduce their independence, such as permanent memory loss (dementia) and definitive forgetfulness. Patients both speaking from experience, as well as those who had not yet experienced these symptoms, stated that such problems may prevent them from performing their professional and daily activities: “*Anything that could affect the brain or ability to concentrate (…) would really be a problem and would mean that I would have to give up my job which I really like and which forms a big part of my life*.” Patients also felt that thinking problems may also result in a change to their identity, and negatively affect how they interact with others. Patients described how difficult it would be to have both thinking problems and mobility problems: “*So then there is not much left because you cannot read a book, (…) you cannot even do something else with your thoughts. And then in fact you can hardly do anything anymore.” One* patient also described how a lack of ability to think—“*a complete loss of thinking*”—makes it difficult to plan anything in the future.

##### Infections

Patients discussed the negative consequences, both physically and mentally, of having an increased susceptibility to infections: “*so we are just afraid of infections, because our resistance is reduced”* (…) especially in view of the COVID-19 pandemic: “*Especially now in the corona crisis, it's not that I'm panicking, but I just keep my distance due to being afraid of infections because our resistance is so low.”* Several specific infections and related problems were described such as lung infections, skin infections/disorders, throat infections, cold, flu, fever, and neutropenia.

##### Reduced Energy

Reduced energy and related problems, including extreme tiredness (also described as exhaustion, fatigue, complete lack of physical strength), sleeping problems, and breathlessness after minimal activity were discussed extensively. Patients described that these problems hinder them from performing daily activities, such as being physically active and independent and hence, significantly reduce their overall quality of life. Mirroring patients' reflections concerning the other side-effects, patients were afraid of permanent and severely reduced energy problems. Further, the psychological burden of having no physical strength and energy was highlighted: “*I'm always a very positive person, but then, my partner was even shocked, that my morale was below zero.”* One patient even mentioned having experienced such severely reduced energy to the extent of losing the ability to see clearly.

##### Pain

Among the several types of pain that MM patients experienced, the most frequent and severe pains patients discussed were: bone pain in the back, chest, feet or hips, muscle pain and cramps, for example in the legs, and nerve pain (sharp, burning, or jabbing pain caused by nerve damage): “*I don't get up without back pain, after a walk I also have back pain.”* The fear of constant and/or more severe pain was repeatedly mentioned, as well as the impact of pain on both the psychological and physical aspects of patients' life's: “*Due to bone pain, many activities are not possible”; “What you have to do to feel less pain is find a posture in which you don't feel it, because of course, it stops you from doing lots of things.”*

Some patients described that there is currently no treatment (including morphine) that alleviates their pain. Similarly, patients hoped for a treatment that would alleviate or eliminate their pain, and thereby help them perform their daily activities and be independent: “*Above all, not feeling pain, when doing any daily activity.”* A life without pain, was considered a (more) normal life: “*To be able to have a normal life, without pain.”* One patient described the unbearable pain experienced due to shingles and post herpetic neuralgia. Some patients also described episodes of headache on the day of treatment, painful urination, and extreme stomach aches following stem cell transplantation: “*Severe stomach ache, I felt like I was on fire from the throat to the rectum.”* One patient, however, mentioned to have never experienced any type of pain: “*I'm atypical, in the sense that I haven't felt any kind of pain.”*

##### Emotional Problems

Patients raised the following emotional problems: (i) easily becoming emotional or becoming less emotional (apathetic); (ii) becoming more aggressive; (iii) feeling depressed; and (iv) feeling insecure because of changes to your body such as: weight loss, weight gain, hair loss, dry eyes, stomach bloating, or abdominal distention—described by one patient as “*9 months pregnant”*—or loss of height due to compressed vertebrae. Patients were afraid of these problems as these often result in personality changes, are daily reminders of their cancer diagnosis and may also prevent patients from doing their daily activities. Several participants found changes to their body problematic as they had a negative impact on their emotional well-being: “*Because, you immediately look different, you don't feel good in your body.”* One patient highlighted that these problems are often considered less important by “outsiders” in comparison to other, life-threatening effects: “*if you tell the doctors that too, and I understand that too, then it is seen as slightly less important.”* It was not clear whether depression and becoming emotional were caused by treatment, the cancer directly, or because of knowing that their life might end soon. For emotional problems as well, the hope for non-lasting, temporary problems as opposed to permanent problems was expressed.

##### Eating and Digestive Problems

Nausea, vomiting, incontinence, constipation, diarrhea, loss of appetite, taste changes, and swallowing problems were all described as problems that significantly reduce patients' quality of life. As for other side-effects and symptoms, several patients were afraid that these problems would become permanent. One patient noted that these problems are problematic because they can lead to reduced social contacts. Whether or not patients had experienced these problems, as well as the severity of the problems they had experienced, depended on patients' particular treatment experience. For example, nausea, diarrhea (and consequently, reduced energy) was linked to treatment with lenalidomide. Some patients noted the burden of retracting gums and jaw problems that prevented them from eating properly and thought these problems were likely due to myeloma rather than treatments.

##### Vision Problems

Patients expressed the fear of suffering from (permanent) vision problems and becoming blind: “*I indeed know a number of people who (…) lost sight. That's a bit of my biggest fear.”* One patient experienced transient vision changes: “*Certainly in the evening, when I am tired, I can hardly see.”* Another patient noted that his vision problems could be due to treatment with lenalidomide. However, it remained largely unclear whether vision problems could be also side-effects of other treatments, due to the cancer itself or perhaps related to aging. For these problems as well, the hope for temporary side-effects as opposed to permanent changes was expressed.

### Other Considerations Relevant for Myeloma Treatment

#### Preferences Differ According to Patients' Individual Characteristics and Experiences

Patients highlighted that their individual preferences were shaped by their previous treatment and disease experience. Particularly, whether they had experienced a certain symptom or side-effect, determined their views and preferences toward those symptoms and side-effects. Patients more frequently raised those symptoms and side-effects they had experienced, heard, or seen with other MM patients than those they or a close contact had never previously experienced: “*When one has gone through these (side-effects), one can think differently from one who hadn't experienced these.”* Whereas, frequent symptoms and side-effects were discussed often, (e.g., bone fractures) others, more new or rare symptoms, were discussed by one or few patients (e.g., vision problems). Aside from treatment and disease experience, it also appeared that age, working status, whether patients have carers (such as children) may be important in determining and understanding why patients place more or less value on certain treatment characteristics. Further, participants who were professionally active frequently emphasized the impact of side-effects that limit their ability to continue working (such as cognitive problems).

##### The Burden of Uncertainty

On several occasions patients discussed the psychological burden of uncertainties including the cause, duration, type, and severity of side-effects and symptoms: “*What can be done about it, is it treatable or does it mean death? And will stopping (the treatment) help (…)? It's a terribly awkward thing.”* Patients also expressed difficulty coping with the uncertainty of the duration of their side-effects and symptoms; patients were afraid that these side-effects would remain permanent or that the side-effects would permanently damage organs: “*So I am also a bit scared; are there no side-effects that are permanent, I am of course also a bit scared, but I still hope that that they really will disappear.”* Regarding uncertainties related to the cause of their problems, patients discussed that at times they we unsure if their symptoms are related to treatment or their myeloma. Some participants stated the importance of managing expectations and that knowing what side-effects to expect before beginning treatment, is important to help them decide whether or not to start or continue a treatment: “*If it is known in advance, then it can be decided that I will not take this treatment because of it.”* Participants also underlined the important role healthcare providers have in addressing these uncertainties: “*Doctors don't say much about these future side-effects (…) In fact, I've had to find out about things myself (…) Maybe [if I had this information] it would have made it easier to accept them and to live with them.”*

##### Hope for New Treatments

Increasing life expectancy was also important to patients as some believed it would increase the chance that during the course of their disease a new, and ideally curative, MM treatment would become available. Patients seemed to be motivated by the knowledge that new treatments are currently being developed and that perhaps one of the novel therapies would become available for them, and in time.

##### Treatability of Side-Effects and Symptoms

Some patients highlighted that when side-effects and symptoms “*can be handled in some way,”* they become manageable and therefore less “important” than side-effects or symptoms for which no treatments are currently available. For example, patients described that severe pains, the development of a new cancer, and cognitive changes are not treatable with current drugs, and therefore perceived as being worse: “*That is, severe pain and the onset of another cancer can really be quite difficult. These problems can be something that can't be helped at all.”*

#### Risk Tolerance Differed Across Participants

Participants seemed to accept that treatments will always have side-effects: “*No matter what the treatment is, side-effects will appear. If I would fear the side-effects I would not be undergoing any treatment.”* Naturally, they hoped for these side-effects to be as few and as mild as possible. However, the severity and number of risks patients were willing to accept in order to receive a certain benefit (i.e., their risk tolerance), differed across patients. Several patients noted that they were willing to accept even severe side-effects if that would be the condition to continue treatment. Others described only to be willing to accept severe side-effects on the condition that the treatment gives noticeable improvements in their disease.

##### Sharing Experiences Among Patients

Participants shared positive feedback regarding their participation in the discussion, they expressed a sense of comfort knowing that other patients experience similar issues. Some expressed that they were happy to be able to have participated in the discussion and share their experiences, feelings, and thoughts with other myeloma patients. The desire to continue the discussion after the focus group discussion was also expressed, as well as the suggestion of gathering via patient support groups.

## Discussion

This study identified treatment and disease-related characteristics (outcomes, side-effects and symptoms) and attribute levels that are key factors in determining treatment attitudes and choices by MM patients. In particular, MM patients across four European countries and with varying disease and treatment experience reached consensus on the importance of the following 11 themes of treatment outcomes, side-effects, and symptoms: life expectancy, life-threatening side-effects, treatment response, mobility problems, thinking problems, infections, reduced energy, pain, emotional problems, eating and digestive problems, and vision problems. Furthermore, this study highlights that MM patients are also concerned with the uncertainties regarding the durability of positive treatment outcomes, as well as the cause, severity and duration of their symptoms and side-effects. Regarding the attribute levels, MM patients feared only short-term positive treatment responses (benefits) but with permanent and severe side-effects and symptoms (risks) such as permanent severe pain or permanent blindness. Finally, this research presents and investigates a specific qualitative methodology in the context of patient preference studies, useful to further the methodological field and enable other researchers to investigate preferences and include results in decision-making that affects patients.

The attributes identified in this research will benefit stakeholders to identify priorities and unmet treatment needs for (new) treatments in MM. Specifically, results from this study point toward a need for MM treatment that not only focuses on extending patients' lives, but as well on improving those symptoms and side-effects that significantly impact MM patients' quality of life. Symptoms and side-effects explained and valued by patients are: mobility problems, thinking problems, increased susceptibility to infections, reduced energy, pain, emotional problems, eating and digestive problems, and vision problems. Furthermore, this research will inform what quality of life-related endpoints and outcomes are important to patients and should therefore be incorporated, in addition to traditional endpoints (such as progression-free survival and overall survival), in MM drug development and evaluation. Examples of HRQoL questionnaires commonly used in myeloma clinical trials are EORTC QLQ-MY2014, FACT-MM, EORTC-QLQ-C30, FACT/GOG-Ntx, and MDASI-MM. Among these, the FACT-MM, EORTC QLQ—MY20, and MDASI-MM are MM specific scales (i.e., including domains specifically related to MM) (50, 51). All of the items included in the MM specific scales were also identified in the current research, which is an important validation of our study results and vice versa, validates the work done to identify the items of these MM-specific scales. However, whereas these scales investigate patients' experience with these problems, the present study also reveals how important these problems are for patients, as well as why they are important and how they impact their lives. Further, this study reveals the following additional specific aspects of importance to MM patients, which are not included in all current MM-specific questionnaires: (i) fear of life-threatening effects, (ii) instability and strange sensations such as hypersensitivity of the skin, numbness or reduced physical sensations, (iii) weakness or stiffness of the legs, feet, toes, and extremities due to nerve damage, (iv) nerve pain, v) the following physical changes: weight loss, stomach bloating, loss of height due to compressed vertebrae, (vi) the following emotional changes: becoming apathetic, aggressive, depression, (vii) eating and digestive problems: such as nausea, vomiting, (viii) vision problems such as blurred vision, (vi) the psychological burden of coping with uncertainties about the durability of positive treatment response, the cause, duration, and severity of side-effects and symptoms.

Some of these additional findings may be explained by the fact that since the development of the HRQoL questionnaires, novel treatments with new side-effects and related uncertainties have been developed and administered to MM patients. For example, life-threatening neurotoxicity's and cytokine release syndrome are new side-effects associated with emerging treatments such as bispecific T-cell engagers and CAR-T therapies, which are not captured by previous questionnaires but in this study were captured in the context of life-threatening side-effects or side-effects that are so significant that they require hospitalization for monitoring (37). Further, (recently) approved drug therapies for MM have been associated with visual changes such as blurred vision and decrease visual acuity (52). The identification of these additional items provides a rationale for including these aspects in a next revision of the HRQoL questionnaires. Systematically including the items identified in this type of research in clinical development, regulatory benefit-risk assessment, HTA/reimbursement decisions and post-marketing decisions, could result in a more patient-centric drug development and evaluation process. Conversely, when there is no evidence that a MM drug targets any of the attributes identified in this study, it may be recommended that such evidence needs to be collected before or after marketing authorization and/or reimbursement and should subsequently be taken into account when designing clinical or real-world evidence research protocols.

This research revealed areas of importance where clear information about MM treatments is needed to inform drug development, regulators, HTA bodies, and healthcare providers. When there is a lack of knowledge and information, e.g., regarding the long-term effects and their severity, this uncertainty should be made public, in an accessibly way to patients. This starts from the clinical trial evidence reported by the pharmaceutical company toward regulators (in clinical trial databases, the marketing application and then reported on the European Public Assessment Report (EPAR), Summary of Product Characteristics (SmPC) and EMA website), and downstream when reporting the clinical trial evidence toward HTA agencies, healthcare providers and patients. MM healthcare professionals, patients, regulators and HTA bodies/payers should be able to easily retrieve this information in clinical trial databases, marketing materials and package inserts of MM drugs. Accurate and clear information about these aspects and uncertainties would result in more informed decision-making by regulators, HTA bodies, physicians and patients. Particularly in the clinical, individual treatment decision-making between healthcare providers and patients, transparent communication before and during treatment may increase patients' satisfaction with the treatment decision and motivation to start or continue a certain treatment and therefore result in better outcomes and patient quality of life as expectations are managed.

Finally, this study may inform the development of PREFER recommendations and future guidance regarding patient preference studies (and methodology) in the context of drug development and evaluation. More specifically, this study derives 10 experience-based learnings regarding the design, conduct and analysis of qualitative research aiming to develop attributes and levels for inclusion in subsequent preference surveys, useful for the PREFER recommendations and future guidance regarding patient preference studies (Table 2).

**Table 2:** Experience-based learnings regarding the design, conduct, and analysis of qualitative research for informing subsequent quantitative preference surveys in the context of drug development and evaluation −10 avenues for optimization.

**Experience-based learnings regarding qualitative research for informing subsequent quantitative preference surveys**
1. **Since patients are disease-experts, experiencing the benefits and risks of treatment on a daily basis, they should be systematically and continuously involved, both as study participants and as study partners**. - The involvement of patients and patient organizations is essential to ensure that the attributes and levels are relevant, comprehensive, and understandable to patients participating in the subsequent quantitative survey. - Their involvement throughout the analysis and attribute selection process guarantees that patients' points of view are reflected in an accurate, unbiased, and understandable way, and thereby improve the survey validity. - In return, patients may benefit from learning about treatments obtained through their involvement. Patient organizations may benefit from using this methodology as an evidence-based way to generate data and best represent the patients' voice. - The results of preference studies may provide patient organizations an evidence-based perspective when communicating with regulatory and reimbursement bodies regarding the priorities and needs of patient communities.
2. **Before undertaking a preference study, researchers should investigate the availability and usefulness of previous preference studies (qualitative or quantitative) for informing the attributes and levels for inclusion in their preference survey**. - If previous studies are available in the disease or treatment context of interest, researchers should assess to what extent the attributes and levels of those studies are transferable and applicable to their research context and aims. This will help determine the necessity of conducting a “new” qualitative study. - In this study, the goal was to identify attributes and levels relevant to patients with varying treatment exposure, disease history, age, or country of origin. This contrasted with previously conducted preference studies identified in our scoping review, which only included patients with a specific disease and treatment experience (e.g., only the relapsed refractory patient population) or used attributes related to a specific treatment. Therefore, a new study considering the recruitment of patients heterogeneous in terms of treatment experience and disease stage was necessary. - Preference researchers aiming to identify attributes and levels relevant to patients with various treatment exposures, disease history, age and country of origin should consider conducting a new qualitative study if a similar qualitative study aiming to pursue this objective is unavailable. - Furthermore, experience from this study highlights that it is desirable: (i) to include a heterogeneous, inclusive sample of patients in terms of treatment exposure and disease history as these variables affected patients' rankings and views, (ii) to include patients from different countries to help ensure a diverse sample of patients is included. - Even if a previous preference study with similar aims is available, preference researchers should assess whether the findings of the study are up-to-date, appropriately designed and comprehensive (i.e., whether they consider novel treatments, as well as related side-effects, outcomes and uncertainties).
3. **Researchers should ensure the study is designed to meet the specific needs of the study participants**. - Key decision points which should be tailored toward the particular patient population of interest are the selection of the qualitative data collection method (the feasibility and usefulness of (telephone) interviews vs. (online) focus group discussions; time, feasibility of ranking exercise) and the development of the questions (via review and pilot testing to ensure relevance, understandability and accuracy). - Input from patients, patient organizations, and/or healthcare providers should help ensure the study is designed in such a way that is easiest for the particular patient population. - In this study, patients, patient organizations, and healthcare providers confirmed that both individual interviews and focus group discussions would be possible and agreed that group interaction would be useful between patients and nominal group technique to trigger discussion around the most important treatment characteristics. In this study, face-to-face discussions were initially planned as myeloma patients are elderly and more likely gravitate away from technology. - However, future researchers may need to balance the utility of increased interaction via focus group discussions vs. the more practical feasibility of individual interviews in view of the targeted participant population. For example, interviews allow for more flexibility in choosing various dates for participation and discussions can take place via telephone and not necessarily online, which is especially relevant in view of COVID19 (and potentially beneficial for elderly patients or those who are not well-versed in technology).
4. **Qualitative studies may also be used to explore which patient variables (such as treatment exposure, disease history, age, or country of origin) should be useful to inform the quantitative survey**. - In this study, patients highlighted that treatment and disease experience strongly influenced their views. - Hence, these variables should be collected and used in qualitative and quantitative preference studies to contextualize both the qualitative and quantitative preference study results.
5. **Obtaining input from stakeholders with expertise in the relevant disease and treatment context (patients, patient organizations, healthcare practioners) and stakeholders with methodological expertise—should help inform the development of attributes and levels**. - Patients can critically reflect on the attributes and levels and thereby avoid inadvertent omittance of attributes and levels of potential importance, as this may bias findings. - Clinicians help ensure the attributes and levels are clinically plausible. - Input from preference research experts helps ensuring the rules of attribute and level development are adhered to.
6. **In view of the multitude of methodological choices in attribute and level development, transparently documenting and describing the study design and methodological choices as well as its limitations and challenges is essential for enabling reviewers to contextualize the study results and evaluate their usefulness for decision-making**.
7. **Before starting a preference study, research teams should investigate the necessity of obtaining ethical approval and contractual agreements with hospitals in all countries where data collection is planned and/or hospitals are involved in data collection, respectively**. - Because ethical approval for this type of research is regulated nationally, researchers should investigate for each country separately, whether the study requires obtaining ethical approval, and if so, consider the time and administrative burden associated with filing and obtaining ethical approval. - Experience from this study reveals that the necessity of obtaining ethical approval depends on whether the study is considered in scope of the national law regulating this type of research. In this study, ethical approval was applied for and obtained in all countries where patients were included. However, during the submission and approval process, it appeared that the study did not fall in the scope of the national law requiring ethical approval in Belgium and Finland, where the process of obtaining ethical approval took particularly long. Conversely, the procedure took less time in Spain and Romania, where the ethical committee did not explicitly mention whether the study required their approval.
**8. Research teams should consider the input, time and administrative burden for involved clinical partners associated with these steps and ensure flexibility in terms of timelines, if ethical approval, hospital contracts, and patient recruitment relies on their cooperation**.
**9. Before starting the study, researchers should investigate how patient recruitment and data collection will take place in practice**. In this study, the involvement of oncology nurses and clinicians proved to be crucial for implementing the recruitment and practical organization of the discussions.
**10. Preference researchers should consider the practical and methodological implications of COVID19 and/or potential subsequent pandemics and how their study designs could best meet patient needs**. - In this study, this was especially relevant as myeloma patients have increased susceptibility to infections. - COVID19 substantially delayed the study, e.g., due to required changes to the initial research protocol to adhere to hospital requirements in view of patient contact restrictions, and increased workloads for cooperating healthcare professionals, ethical committees and hospital administration offices. - Future qualitative preference research may likely require digital and online formats for data collection, as well as phone calls, virtual encounters, instead of face-to-face contacts.

If preference studies are to inform drug development, regulatory, and reimbursement decisions, it is essential to reflect on how the key attributes and levels for inclusion in preference survey were identified. Misspecification of attributes may lead to biased findings, and hence, biased preference studies, hence undermining development, regulatory, and reimbursement decisions. It is therefore important to reflect on how the characteristics identified in this study compare to those identified in previous preference studies among MM patients. Comparing the attributes found in this study to those identified in the scoping review of previous preference studies (Appendix 2), reveals that a large portion of these studies used attributes that were not appointed by patients themselves but developed using top-down methods, starting from the perspective of researchers, developers or decision-makers. Previous preference studies have also used attributes that were associated with one specific therapy. In contrast, this study identified patient-relevant attributes across different therapies (for example, novel immunotherapies), countries and directly from myeloma patients. There are several potential reasons for differences in attributes identified across preference studies. Attribute identification, to date, is mostly done through studies involving literature reviews and qualitative empirical studies. Qualitative empirical research always requires contextualizing the results in view of the research setting. This implies that several factors may differ across qualitative studies, such as the selected sample, the stakeholder conducting study (e.g., a patient organization vs. a pharmaceutical company), the researcher or decision-makers' interests, the time of study and the specific questions asked. All of these factors need to be taken into account when looking at the results (i.e., the identified attributes) as a difference in any of these may already explain a difference in the identified attributes. Differences in methodology for attribute and level identification are likely triggered by uncertainties regarding the best methodological approach for this study type. While the present study derived experience-based learnings, the methodological field is continuously and rapidly evolving, and other qualitative study methods are also under investigation. Combining and comparing experiences and methodological understanding from different qualitative approaches will be useful to inform the development of a standardized approach for use by all stakeholders across disease areas. Furthermore, methodological understanding will assist with the development of a validated framework for designing and conducting preference studies aiming to inform drug development and evaluation.

Although the attributes reflect areas of consensus, there was heterogeneity with regards to the value each patient attached to the attributes. In particular, participants more frequently valued those symptoms and side-effects they had previously experienced. Further, participants who were older, had undergone more treatments, or had no young children seemed to attach more importance to quality of life related attributes (such as pain) than life expectancy, and vice versa. Participants who were professionally active frequently emphasized the impact of (cognitive) side-effects on their ability to continue working. Likewise, participants' willingness to accept more reduction in quality of life (i.e., symptoms and risk of life-threatening side-effects) in return for a potential increase in life expectancy differed across participants, depending on their individual situation and personal attitudes. These findings underscore the importance of further quantitative preference research that statistically substantiates these hypotheses and provides a quantified understanding of individual patients' values of life extension vs. symptom reduction vs. risk of life-threatening side-effects.

The existence of patient subgroups with systematically different preferences may be viewed both as a challenge and opportunity from the perspective of decision-makers (industry, drug developers, and HTA bodies) who develop, authorize and reimburse drugs for groups of patients and not for individual patients. In particular, it raises the question whether their decisions need to be tailored toward specific patient populations whose preferences align with the product characteristics being developed or evaluated. The existence of subgroups in the MM patient population with systematically different relative attribute values and risk tolerances may also inform the identification of key areas of unmet needs, benefits and risks for this relevant population. For example, a company could submit clinical evidence to apply for marketing authorization and reimbursement for the elderly, more treatment experienced MM population (also called the relapsed refractory RRMM) and clinical trial results may indicate that the treatment causes quality of life related problems (such as mobility, vision problems) in this population. If results from a preference study reveal that this population finds quality of life related attributes (such as mobility, vision problems) more important than life expectancy, then decision-makers should likely place more value on these risks during their assessment of treatment outcomes and ensure these risks are taken into account during their decision-making.

As for the limitations, it is important to reflect on the impact of the COVID-19 on this research. This is especially relevant since this study consisted of qualitative discussions with MM patients, who have a higher susceptibility to infections. Conducting this study during COVID-19 required flexibility from both participants and the study team. For the online and telephone discussions, it is likely that participants, who were not comfortable with online discussions or telephone (e.g., older participants), were less likely to participate. The study team tried to be as inclusive as possible during recruitment, by offering both face-to-face and online discussions, according to the preferences of the participants and the local social distancing and hospital guidelines. Further, technical support for participants was given throughout the entire study. A steps-wise guideline explaining the practicalities of the discussion beforehand, and ensuring there was an opportunity, before the session, for participants to test whether they could participate in the discussion. Still, the median age of diagnosis of patients included in this study was 55, which is 11 years younger than the median age reported by Kazandijan in 2016 and 14 years younger than the average age of diagnosis reported by ASCO in 2020 (53, 54). However, there was a large age range between the youngest and oldest patient (46–73), and therefore the attributes captured in this study for inclusion in the next quantitative phase also reflect those that are most important for elderly patients. Further regarding generalizability, it is important to note that the purpose of this study was not to make statements about a population larger than the included sample. Rather, it aimed to gain in-depth insight into the opinions of patients participating in the discussions including which attributes are important to them and why.

Participant heterogeneity in terms of treatment and disease experience [including the type of treatments received, disease stage (i.e., refractory level and number of previous treatments, experienced side-effects)] and demographic characteristics were introduced to avoid distortions in the data; it was envisioned that these personal characteristics and experiences could influence participants' opinions. Hence, heterogeneity among the focus group participants, particularly regarding their disease and treatment experience, triggered interactions between patients with varying backgrounds and facilitated discussions around a large range of symptoms, treatment outcomes, and side-effects, even though some individual participants (earlier diagnosed, with less treatment experience) had not experienced them themselves. The inclusion of a heterogeneous group of participants and their interactions, due to the focus group methodology, helped ensure that the identified attributes and levels are not geared to only patients with a particular background (e.g., with a particular therapy experience, disease experience, level of education). Likewise, to ensure that patients would be able to discuss “future” treatment outcomes and side-effects of novel treatments or treatments under development (i.e., symptoms they had not yet experienced themselves, such as those associated with bispecific T-cell engagers and CAR-T), the focus group discussions included endpoints and adverse events reported in phase 3 MM clinical trials from 2010 onwards (found in the literature review). Further, the attribute selection also captured the favorable and unfavorable effects of (recently) approved MM drug therapies.

The results of this (and other qualitative research) are time, study context and participant bound and hence need to be interpreted considering the specific time period and (drug therapy) context the study took place as well as in view of the type of participants that took part. For example, this study took place during COVID19 and this may have led to the fact that patients more frequently raised their increased susceptibility to infections. Further, the results should be viewed in the specific drug therapy context patients are experiencing currently; the MM treatment context is rapidly evolving and a significant number of new and emerging treatments have been introduced. These novel treatments are bringing prolonged survival but also potential side-effects of uncertain severities and duration on the long term. For example, new treatments such as novel immunotherapies (e.g., bispecific T-cell engagers and CAR-T) have been associated with (acute) side-effects and these may cause psychological and physical distress to patients. The introduction of these new therapies and rapidly evolving drug therapy context likely explains why patients expressed the psychological burden of their uncertainties related to their side-effects and efficacy outcomes, particularly regarding treatments only being marketed and prescribed for a relatively short time period. This study highlights that patients are concerned with uncertainties regarding the long-term duration and severity of their side-effects (such as neuropathic, mobility and vision problems) and about how long the treatment will continue to work for them. Information about which uncertainties are most important to patients may help stakeholders (drug developers, regulators, HTA bodies, physicians) by providing insights about the uncertainties most pressing to patients; to be considered during decisions about evidence generation, marketing authorization, market access and subsequently managed in the individual treatment decision-making context.

Several patient characteristics (disease stage such as refractory level and number of previous treatments), symptom experience, and demographic data (including country of origin, health literacy/education) likely influence preferences, i.e., the value that participant place on attributes (outcomes, symptoms, and side-effects). For example, MM patients that are younger, less frail and who have limited treatment exposure may tolerate and perceive side effects differently. Therefore, it will be important to investigate, transparently describe, and consider the impact of these patient variables on preferences, and to describe this impact as well as their influence on preferences. Therefore, in the survey following this qualitative study, we aim to characterize and describe the demographics of the study population using patient characteristics when reporting the results. Additionally, in the follow up quantitative survey, research will focus on the statistically significant impacts of patient characteristics on preferences.

## Conclusion

This study gained an in-depth understanding of the treatment and disease-related characteristics (outcomes, side-effects, and symptoms) and types of attribute levels (severity, duration) that are most important to MM patients. Results point toward a need for MM drug development, evaluation, and individual treatment decision-making that not only focuses on extending patients' lives, but that addresses symptoms and side-effects that significantly impact MM patients' quality of life. This study underlines the need for communication toward patients about the short and long-term effects of MM treatments. Finally, this study may help stakeholders to understand which quality of life-related treatment outcomes are most important to MM patients and therefore should be considered for systematic incorporation in MM drug development, evaluation and clinical practice.

## Data Availability Statement

The datasets presented in this article are not readily available because they contain information that could compromise participants' privacy and consent. Requests to access the datasets should be directed to rosanne.janssens@kuleuven.be.

## Ethics Statement

The studies involving human participants were reviewed and approved by the Ethics Committee UZ/KU Leuven (reference S63620), the Clinical Institute Fundeni (reference 20637), the Research Ethics Committee of the Bellvitge University Hospital reference PR117/20 (CSI 20/26), and the Helsinki University Hospital (reference 1413/2020). The patients/participants provided their written informed consent to participate in this study.

## Author Contributions

RJ drafted the manuscript. All authors provided substantial input in the study design, critical revision of the manuscript, and read and approved the final manuscript.

## Conflict of Interest

The authors declare that the research was conducted in the absence of any commercial or financial relationships that could be construed as a potential conflict of interest.
