# Supplementary material for: Patient Preferences for Multiple Myeloma Treatments: A Multinational Qualitative Study
Source: Front Med (Lausanne). 2021 Jul 6;8:686165. doi: 10.3389/fmed.2021.686165 (PMC8289885; doi:10.3389/fmed.2021.686165)
Supplement: Supplementary file 1 [file Data_Sheet_1.PDF]

Supplementary Material

Patient Preferences for Multiple Myeloma Treatments: A Multinational Qualitative Study

Rosanne Janssens[1], Tamika Lang[2], Ana Vallejo[2], Jayne Galinsky[2], Ananda Plate[2], Kate Morgan[2], Elena Cabezudo[3], Raija Silvennoinen[4,5], Daniel Coriu[6,7], Sorina Badelita[7], Ruxandra Irimia[6,7], Minna Anttonen[8], Riikka-Leena Manninen[8], Elise Schoefs[1], Martina Vandebroek[9], Anneleen Vanhellemont[10], Michel Delforge[10], Hilde Stevens[11], Steven Simoens[1], Isabelle Huys[1]

[1]Department of Pharmaceutical and Pharmacological Sciences, KU Leuven, Leuven, Belgium

[2]Myeloma Patients Europe, Brussels, Belgium

[3]Department of Haematology, H. Moises Broggi / ICO-Hospitalet, Barcelona, Spain

[4]Department of Hematology; Helsinki University Hospital Comprehensive Cancer Center

[5]University of Helsinki, Helsinki, Finland

[6] "Carol Davila" University of Medicine and Pharmacy, Bucharest, Romania

[7]Fundeni Clinical Institute, Bucharest, Romania

[8]Association of Cancer Patients in Finland, Helsinki, Finland

[9]Faculty of Economics and Business, KU Leuven, Leuven, Belgium

[10]University Hospital Leuven, Leuven, Belgium

[11]Institute for Interdisciplinary Innovation in healthcare (I3h), Université Libre de Bruxelles (ULB), Brussels, Belgium

*** Correspondence:**
Rosanne Janssens
rosanne.janssens@kuleuven.be

Appendix 1: Answer sheet containing ranking exercise

Dear participant,

Please fill out the information below. We would like to collect your answers on the questions below to learn more about you as a focus group participant. The answers of all participants on these questions will be summarized as group characteristics in reports and publications on this focus group. All information provided by you will be "pseudonimised", this means that the results of this study will never be linked to your name and identity.

If something in this answer sheet is not clear to you, please do not hesitate to contact *(placeholder contact person)*.

<u>Section 1: Personal characteristics</u>

1. What is your age?

2. What is your gender?
   a. Female
   b. Male

3. Which country or countries are you a citizen of?

4. What is the highest level of education that you completed?
   a. No diploma
   b. Primary education
   c. Secondary education (high school)
   d. Bachelor degree (college or university)
   e. Master degree (university)
   f. PhD (university)
   g. I prefer not to answer this question
   h. Other, please specify:

5. Regarding your activity and physical function, how would you generally rate your activity over the past month[1]:
   a. Normal with no limitations
   b. Not my normal self, but able to be up and about with fairly normal activities
   c. Not feeling up to most of the things, but in bed or chair less than half the day
   d. Able to do little activity and spend most of the day in bed or chair
   e. Pretty much bed ridden, rarely out of bed

---

[1] *PG-SGA/Pt-Global Platform: pt global; 2014 [Available from: http://pt-global.org] (Copyright FD Ottery)*

6. What is your current living situation?
    a. I live alone
    b. I don’t live alone (e.g. living with a partner, family, friends, …)
    c. I prefer not to answer this question

7. What is your work status?
    a. Student
    b. Employed (full-time, part-time, ...)
    c. Unemployed / Looking for work
    d. Retired
    e. Other
    f. I prefer not to answer this question

8. At what age were you diagnosed with multiple myeloma?

9. Which of the following best describes best your current treatment situation:
    a. I am newly-diagnosed and do not require treatment
    b. I require treatment but the treatment has not yet started
    c. I am in remission (= a decrease in or disappearance of signs and symptoms of cancer)
    d. I am currently receiving treatment
    e. I am unsure
    f. I prefer not to answer

10. If you are currently receiving treatment, what treatment are you taking?

11. If you are currently receiving treatment, how many treatment lines have you received (the current one included)? One treatment line of treatment refers to one drug or a combination of drugs that are usually given for a specific time duration.

12. If you are not currently taking medication, what is the reason for this?

13. If you have already undergone treatment, please describe what was/were the worst side-effect(s) you experienced?

14. Are you currently enrolled in a clinical trial (a research study performed in people that are aimed at evaluating a medical, surgical, or behavioral intervention)?
    a. Yes
    b. No, but I have been in the past
    c. No, and I have never been in the past

15. Are you in regular contact with other patients of a patient organization support group?
    a. Yes
        a. If yes, please specify what your contact was (e.g. presentation, gathering, workshop) and how frequent:
    b. No
    c. Prefer not to answer

16. Do you have other chronic (long-lasting) health problems (e.g. other types of cancer, arthritis, depression, diabetes) that require you taking drugs or receive other types of medical treatment
    a. Yes, namely:
    b. No
    c. Prefer not to answer

17. How often do you have someone (like a family member, friend, hospital/clinic worker, or caregiver) help you read hospital materials?
    a. Always
    b. Often
    c. Sometimes
    d. Occasionally
    e. Never

18. How often do you have problems learning about your medical condition because of difficulty understanding written information?
    a. Always
    b. Often
    c. Sometimes
    d. Occasionally
    e. Never

19. How confident are you filling out medical forms by yourself?
    a. Extremely
    b. Quite a bit
    c. Somewhat
    d. A little bit
    e. Not at all

Section 2: Identifying treatment characteristics that matter most to you

1. *When you undergo a treatment for multiple myeloma, what* ***improvement*** *do you expect from it? With improvement we mean benefits, favorable or desirable effects. Please also explain* ***why***

____
____
____
____
____

2. *Multiple myeloma treatments may also be associated with side-effects. With side-effect we mean risks or undesirable effects of the treatment. Imagine you would start a certain treatment, what* ***side-effects*** *would make you want to doubt whether you want to start taking it? Please also explain* ***why***

____
____
____
____
____

3. *Imagine you have started taking a certain treatment, what* ***improvements*** *would make you want to accept more of the side-effects you listed? Please also explain* ***why***

____
____
____
____
____

4. *Imagine you have started taking a certain treatment, what* ***side-effects*** *would make you want to reconsider whether you want to continue the treatment? Please also explain* ***why***

____
____
____
____
____

Section 3: Grading of treatment characteristics

1. Please carefully read the below characteristics and their explanations. These characteristics can be both positive and negative effects and are related to multiple myeloma or multiple myeloma treatments. These characteristics can be:

- Symptoms of multiple myeloma
- Positive effects or negative effects (side effects) of treatments that are already being prescribed to patients
- Potential future positive or negative effects (side effects) of treatments that are currently being developed and not yet prescribed to patients

The chance these characteristics may occur in a specific patient may be very different for each of the characteristics; some are side effects of specific treatments and may occur very rarely while others more frequently.

The chance these effects occur in a specific patient may depend on different factors (such as the current treatment, disease history, patient characteristics…).

2. If you find that one or more treatment characteristic (s) that you entered in the previous section 2 are missing, please add this / these characteristic (s) to the end of the list under "Missing treatment characteristics". Please also include a description for that characteristic.

3. Place a number after each treatment characteristic in the "Rating" column to indicate how important it is to you on a scale from 1 to 5:

1 = not important at all

2 = not important

3 = neutral

4 = important

5 = very important

Do not hesitate to ask questions to *(placeholder contact person)* if you do not understand something. Your opinion of these characteristics will be asked during the discussion, so it is important that you understand what they mean.

Characteristic	Explanation	Grading
Arrhythmia	- The heartbeat is irregular, too fast, or too slow.<br>- This can occur in up to 6% of myeloma patients[2].	
Bleeding	- Abnormal bleeding, for example when brushing your teeth, (spontaneous) bruising, or the presence of blood in urine or feces.<br>- This can occur in up to 62% of myeloma patients.	
Bone or back pain	- This can occur in up to 23% of myeloma patients.	
Cancer	- New cancer, e.g. cancer of the bladder, the blood cells, intestines.<br>- This can occur in up to 10% of myeloma patients.	
Chest pain	- This can occur in up to 11% of myeloma patients.	
Fever	- A temperature above 38 degrees.<br>- This can occur in up to 40% of myeloma patients.	
Nausea	- Feeling of sickness or discomfort in the stomach that may come with an urge to vomit.<br>- This can occur in up to 65% of myeloma patients.	
Headache	- This can occur in up to 30% of myeloma patients.	
Rash	- Reddish discoloration of the skin.<br>- This can occur in up to 42% of myeloma patients.	
High or low blood pressure	- A high blood pressure is blood pressure reading higher than 140 millimeters of mercury (mm Hg) for the top number (this is the upper pressure) and/or 90 mm Hg for the low number (this is the under pressure).<br>- A low blood pressure is blood pressure reading lower than 90 mm Hg for the top number (upper pressure).<br>- This can occur in up to 14% of myeloma patients.	
Coughing, wheezing, and shortness of breath	- Wheezing is a high-pitched whistling sound made when you breathe.<br>- May occur as a result of damage to the lungs.<br>- This can occur in up to 35% of myeloma patients.	
Fatigue	- Extreme tiredness and inability to function due to lack of energy.<br>- May be acute (short and intense) or chronic (longer lasting).	

[2] As the grading of treatment characteristics may also depend on the frequency by which these may occur, the list of treatment characteristics also included a description of how frequent the effects presented may occur in real life (Appendix 1). For (novel) MM treatments already being prescribed, the frequencies were extracted from the following sources: [1-9]. For MM treatments currently in development, the following sources were used: [10, 11].

	- This can occur in up to 56% of myeloma patients.	
Insomnia	- Lacking sleep or difficulty falling asleep.<br>- This can occur in up to 30% of myeloma patients.	
Edema	- Swelling caused by excess fluid in body tissues.<br>- This can occur in up to 25% of myeloma patients.	
Heart failure	- Failure of the heart to pump the blood to all body tissue.<br>- Can give rise to edema, shortness of breath, fatigue, arrhythmia and persistent coughing or wheezing.<br>- This can occur in up to 4% of myeloma patients.	
Infections	- Growth of microbes in the body.<br>- This can occur in up to 80% of myeloma patients.	
Thinking impairment	- Slowed thinking and processing of information.<br>- This is a neurological effect. Neurological effects together can occur in up to 42% of myeloma patients.	
Memory loss or amnesia	- This is a neurological effect. Neurological effects together can occur in up to 42% of myeloma patients.	
Hallucination	- This is a neurological effect. Neurological effects together can occur in up to 42% of myeloma patients.	
Lower level of consciousness	- This is a neurological effect. Neurological effects together can occur in up to 42% of myeloma patients.	
Confusion	- This is a neurological effect. Neurological effects together can occur in up to 42% of myeloma patients.	
Dizziness	- This is a neurological effect. Neurological effects together can occur in up to 42% of myeloma patients.	
Tingling	- Sensation of slight prickles or stings in the skin.<br>- This is a neurological effect. Neurological effects together can occur in up to 42% of myeloma patients.	
Neuropathic pain	- Pain to the nerves.<br>- This is a neurological effect. Neurological effects together can occur in up to 42% of myeloma patients.	
Numbness of the skin	- This is a neurological effect. Neurological effects together can occur in up to 42% of myeloma patients.	
Change in psychological and mental status	- Changes in how patients think, feel, their moods, beliefs, ways of coping, and relations with family, friends and co-workers.<br>- No data was found on how frequent this may occur.	

Quality of life	- A multi-dimensional concept that includes domains related to physical, mental, emotional, and social functioning. It focuses on the overall health status.	
Response to treatment	- How well a patient responds to treatment, based on whether there is a reduction in the signs of cancer.	
Loss of body weight	- Sometimes associated with swallowing difficulties and throat pain.<br>- This can occur in up to 45% of myeloma patients.	
Decreased appetite	- This can occur in up to 45% of myeloma patients.	
Muscle weakness or cramps/spasms	- This can occur in up to 15% of myeloma patients.	
Destructions of the bone and bone fractures	- This can occur in up to 80% of myeloma patients.	
Tremor	- Involuntary, rhythmic shaking movements<br>- This can occur in up to 30% of myeloma patients.	
Sepsis	- The body reacts extremely in response to an infection. This leads to low blood pressure and other abnormalities in the body.<br>- No data was found on how frequent this may occur.	
Diarrhea	- This can occur in up to 55% of myeloma patients.	
Constipation	- This can occur in up to 45% of myeloma patients.	
Stroke	- Brain cells die because of lack of oxygen<br>- Usually caused by a blood clot that blocks or plugs a blood vessel in the brain.<br>- This can occur in up to 3% of myeloma patients.	
Progression free survival	- The length of time during and after the treatment that a patient lives with the disease but it does not get worse.	
Overall survival	- The length of time from either the date of diagnosis or the start of treatment that patients diagnosed with the disease are still alive.	
**Missing treatment characteristics**	**Explanation**	**Grading**
	-	

	-	
	-	
	-	
	-	
	-	
	-	

Please provide your completed answer form to ***(placeholder contact person)***

Appendix 2: Published patient preference studies in Multiple Myeloma

Date of search: 25 March 2019 – updated 26 March 2021

Databases used: Pubmed and Embase

Selection based upon title and abstract

All publication dates

Inclusion criteria:

- Preferences from MM patients

Exclusion criteria:

- If preferences not from patients (only caregivers, clinicians)
- If no preference method (qualitative/quantitative) was applied[3]
- If no preferences reported
- Study protocols
- If preferences elicited *only* for non-medical product-related treatment characteristics (e.g. for participation in decision-making, for remote monitoring, for information, physical activity)
- If already included via other database

Search query Pubmed:

(preference[Title/Abstract] OR preference'[Title/Abstract] OR preference's[Title/Abstract] OR preferencec[Title/Abstract] OR preferenced[Title/Abstract] OR preferencefor[Title/Abstract] OR preferencel[Title/Abstract] OR preferenceperformance[Title/Abstract] OR preferences[Title/Abstract] OR preferences'[Title/Abstract] OR preferences5ethical[Title/Abstract] OR preferencesin[Title/Abstract] OR preferencesrd[Title/Abstract] OR preferencetrade[Title/Abstract] OR preferencextraining[Title/Abstract]) AND multiple myeloma[Title/Abstract]

---

[3] *Following preference methods were considered:*

- *Preference exploration methods:* ***individual techniques*** *(interviews, complaint procedures),* ***group techniques*** *(Delphi method, focus group, public meeting, nominal group technique, citizens' juries) and* ***concept mapping***
- *Preference elicitation methods:* ***discrete-choice based related techniques*** *(discrete choice experiment/conjoint analysis, best-worst scaling type 3, self-explicated conjoint, measure of value),* ***threshold related techniques*** *(standard gamble, time trade-off, person trade-off, starting known efficacy, test trade-off, threshold technique, contingent valuation),* ***rating related techniques*** *(constant sum scaling, repertory grid method, analytic hierarchy process, swing weighting, visual analogue scale, allocation of points, outcome prioritization tool) and* ***ranking related techniques*** *(qualitative discrimination process, q-methodology, control preferences scale, best-worst scaling type 1,2)*

Search results Pubmed:

Results Pubmed:

75 results, 10 included

Search query Embase:

'preference*':ab,ti AND 'multiple myeloma':ab,ti

Results Embase:

175 results, 4 included

<u>Combined results (n=15)</u>

First author, year	Type of publication	Research objective	Sample	Preference method(s)	Attributes/items/criteria	Results
Blade, 2018	Journal article	To define a standard set of outcomes and the most appropriate instruments to measure them for managing newly diagnosed patients with multiple myeloma (MM).	Haemato-<br><br>logists, hospital pharmacists and patients	Delphi method	NA	Consensus to use OS, PFS, minimal residual disease and treatment response to assess survival and disease control. Agreement to measure health-related quality of life, pain, performance status, fatigue, psychosocial status, symptoms, self-perception on body image, sexuality and preferences/satisfaction
Lassalle, 2016	Journal article	To evaluate home versus hospital administration of s.c. bortezomib with respect to patient preference and cost	50 MM patients	2 questionnaires analyzing criteria (Likert scale)	16 criteria, such as quality of life, well-being, social life, satisfaction,	The evaluation of patient preference and satisfaction showed that home administration improved the quality of life in 84% of the patients, increased well-being in 78%, and improved the activities of daily living in 72% of the cases. Overall, 98% of the patients noted their preference for home

					safety, quality of care, the reduction in personal transportation time, and personal anxiety	administration over the hospital administration of bortezomib.
Ludwig, 1997	Journal article	Investigating PP for interferon alfa in MM	355 US MM patients	phone interviews	NA	Approximately half of the patients accepted the unidentified treatment if remission and/or survival improved by at least 6 months. Accepters were younger and more likely to have used interferon. Of patients who rejected the unidentified treatment, 25% to 50% would have been willing to accept it if the benefits were > or = 12 months. Test/retest reliability of all choices, determined in 36 cancer patients, was 0.896
Monterosso, 2018	Journal article	To describe the unmet informational, psychological, emotional, social, practical, and physical needs and preferences for posttreatment survivorship care of individuals living with multiple myeloma to	2 x 14 MM patients	2 FGDs	NA	Thematic analysis revealed 7 key themes: information needs, experience with health-care professionals, coping with side effects, communicating with family and friends, dealing with emotions, support needs, and living with the chronicity of myeloma. Participants described key characteristics of survivorship care relevant to their needs and indicated they would like a more whole of person approach to follow-up when the main treatment phases had completed.

		inform the development of relevant, person-centered, survivorship services.				
Mühlbacher, 2008	Journal article	To explore patients' preferences regarding treatment of MM	3 x FGD with 6-8 MM patients each; 282 patients in survey	1) 3 FGD with MM patients, 2) Survey using direct assessment and a DCE	Life expectance, symptoms, relation to other therapies, adverse reactions, duration of adverse reactions, dosage therapy burden, drug absorption, self-related role in therapy, therapy setting, presence of the disease, financial involvement, physical ability, physical feeling, emotional status, time perspective, social	Direct measurement showed effectiveness aspects (i.e. high effectiveness, long lasting effects, max. prolonged life expectancy) and further treatment options in the first places, followed by maximal prolonged life expectancy, minor side effects and therapy-free-intervals. In the DCE, alternatives with further treatment options, longer life expectancy, "not always think of the disease" and therapy-free-intervals were more likely to be chosen, giving thus similar results.

					life activities	
Osborne, 2014	Journal article	To: (1) explore the issues important to QOL from the perspective of people with multiple myeloma, and (2) explore the views of patients and clinical staff on existing QOL questionnaires and their use in clinical practice	MM patients, clinical staff	2 types of semi-structured qualitative interviews, 3 FGDs	NA	Main themes important to QOL were Biological Status, Treatment Factors, Symptoms Status, Activity & Participation, Emotional Status, Support Factors, Expectations, Adaptation & Coping and Spirituality. Symptoms had an indirect effect on QOL, only affecting overall QOL if they impacted upon Activity & Participation, Emotional Status or Support Factors. This indirect relationship has implications for the design of QOL questionnaires, which often focus on symptom status. Health-service factors emerged as important but are often absent from QOL questionnaires. Sexual function was important to patients and difficult for clinicians to discuss, so inclusion in clinical QOL tools may flag hidden problems and facilitate better care. Patients and staff expressed preferences for questionnaires to be no more than 2 pages long and to include a mixture of structured and open questions to focus the goals of care on what is most important to patients.
Postmus, 2018	Journal article	To elicit the preferences of patients with multiple myeloma regarding the possible benefits and risks of	560 MM patients	Online survey based on multicriteria decision analysis and swing	(a) 1-year progression-free survival, (b) mild or moderate toxicity	The average weight given to PFS was 0.54, followed by 0.32 for severe or life-threatening toxicity and 0.14 for mild or moderate chronic toxicity. Participants who ranked severe or life-threatening toxicity above mild or moderate chronic

		cancer treatments and to illustrate how such data may be used to estimate patients' acceptance of new treatments.		weighting. FGD with patients and clinicians to identify attributes.	for 2 months or longer and (c) severe or life-threatening toxicity	toxicity (56%) were more frequently younger, working, and looking after dependent family members and had more frequently experienced severe or life-threatening side effects. The amount of weight given to PFS did not depend on any of the collected covariates. The feasibility of using the collected preference data to estimate the patients' acceptance of specific multiple myeloma treatments was demonstrated in a subsequent decision analysis example.
Rowen, 2011	Journal article	To estimate a preference-based measure for cancer from the EORTC QLQ-C30	350 members of UK population	Time trade-off	physical functioning, role functioning, social functioning, emotional functioning, pain, fatigue and sleep disturbance, nausea, constipation, and diarrhea	The health state classification system has eight dimensions (physical functioning, role functioning, social functioning, emotional functioning, pain, fatigue and sleep disturbance, nausea, constipation, and diarrhea) with four or five levels each. Regression models have few inconsistencies (0 to 2) in estimated preference weights and small mean absolute error ranges (0.046 to 0.054).
Tariman, 2014	Journal article	To examine patient perspectives on their personal and contextual factors relevant to treatment	MM patients > 60 years, newly diagnosed, and physicians directly	Semi-structured interviews	NA	Themes related to treatment decision making among patient participants include various decisional role preferences; several sources of information related to myeloma; contextual and patient-specific factors influence treatment decisions; negative perceptions related to

		decision-making	providing care to MM patient participants			the treatment decision-making process exist; strong desire to be in remission and to live a longer life; For physician participants, top themes related to decision making were: QOL or survival considerations or simultaneously considerations of treatment effectiveness, QOL and survival; screening patients for eligibility for autologous HSCT; time is a barrier to effective TDM; Various methods were used to assess patient decisional role preferences.
Wilke, 2018	Journal article	To describe preferences of with novel proteasome inhibitor-based combination treatments	84 German relapsed refractory multiple myeloma (RRMM) patients	DCE developed upon literature and 2 FGDs with patients	"therapy application regimen," "time without progression of disease," "possibility of grade >/=3 adverse events (AEs) affecting the blood," and "possibility of grade >/=3 AE heart failure."	Among the tested attributes, "therapy application regimen" was assigned the highest importance for treatment decisions (38.8%), the second important attribute was "time without progression of disease" (38.7%), followed by "possibility of AE heart failure" (13.9%) and "possibility of AEs affecting the blood" (8.6%). Patients preferred oral intake once a day and once a week over other application modes such as oral intake once a day and once a week plus twice-weekly infusions. Furthermore, they preferred longer disease progression-free time and lower risk of grade >/=3 AEs. The highest overall utility was derived for ixazomib + lenalidomide + dexamethasone (utility: 3.218), compared with lenalidomide + dexamethasone (2.769), and carfilzomib + lenalidomide + dexamethasone (1.928).

Auclair, 2017	Conference abstract	To quantify PP to elicit treatment priorities and unmet needs.	Patients (anticipated 150) and caregivers (anticipated 150) participating in the Multiple Myeloma Research Foundation CoMMpass study	DCE survey coupled with a best-worst scaling (BWS) exercise. A literature review, focus groups, and consultation with research team members to identify key treatment attributes and levels.	PFS, risk of heart failure, peripheral neuropathy, risk of low blood counts, combining thrombocytopenia and neutropenia, gastrointestinal problems, and mode and frequency of administration	Respondent comments from pretests suggest PFS remains a very important attribute to patients and their caregivers.
Bauer, 2017	Conference abstract	To elucidate patient preferences regarding different novel treatment options for relapsed refractory multiple myeloma (RRMM)	RRMM patients	interviews and DCE	Drug administration, time without disease progression, possibility of side effects affecting the blood, possibility of heart failure	Drug administration was the most important attribute for patients' choices (relative importance 38.83%). Patients strongly preferred application 1 (utility: 1.79; $p < 0.001$), followed by application 2 (1.46; $p < 0.001$), both compared to application 3. The second most important attribute was disease-progression- free time (38.63%) with utilities of 1.78 for 26 months ($p < 0.001$) and 0.81 for 20 months ($p < 0.001$), both compared to 17 months. Possibility of heart failure occurrence had an importance of 13.92% (utility of 2% vs. 4%: 0.64; $p < 0.001$), and possibility of side

						effects affecting the blood had the lowest importance (8.62%; utility of 12% vs. 19%: 0.40; p< 0.001). Derived utilities for currently available RRMM treatment options were 3.21 for Ixazomib+Lenalidomide+Dexamethasone, 2.75 for Lenalidomide+Dexamethasone and 1.89 for Carfilzomib+Lenalidomide+Dexamet hasone.
Leleu, 2018	Conference abstract	To understand MM patients' strength of preference for method of administration and for avoiding specific adverse events (AEs)	400 MM patients from 8 countries: Canada, Denmark, France, Germany, Italy, Spain, Sweden, UK	Online DCE	AEs (bone pain, febrile neutropenia, hypokalemia, hyponatremia, infection, lymphopenia, neuralgia, neutropenia, peripheral neuropathy, renal adverse reaction, and thrombocytopenia and thromboembolic events), route of administration, and PFS	Patients showed a preference for oral vs IV administration and there was a trend toward preferring oral over SC administration. Strength of preference declined in patients with prior treatments. Patients expressed a statistically significant preference to avoid all presented grade 3/4 AEs, except for hematologic AEs: thrombocytopenia, neutropenia, and lymphopenia for first treatment patients, and neutropenia for patients with prior therapy. The relative importance of bone pain, infection, and thromboembolic events was lower in patients with prior therapies, while the relative importance of grade 3/4 neuralgia, febrile neutropenia, and renal adverse reaction increased.

McKay, 2018	Conference abstract	To describe patient reported preferences within the context of currently available MM treatments	Interview with MM patients, survey to adults diagnosed with MM, who had received or were currently receiving first-line therapy (FL), second-line therapy (1PL), or third-line therapy (2PL) at the time of the survey (target N=200).	Qualitative phase using semi-structured interviews and quantitative phase using DCE online survey. Treatment attributes and levels were identified through literature review, current treatment guidelines, and clinical input.	overall survival (OS), progression free survival (PFS), dosing, and tolerability were identified in qualitative phase	Results from this interim analysis suggest that patient preferences for MM treatments may vary by treatment history. Additionally, when efficacy is similar, a significant number of patients place greater importance on dosing frequency than on the duration of treatment administration. Patients may consider treatment options holistically - e.g., convenience is not simply 'chair-time,' but rather also includes frequency of outpatient visits. This study provides insight into how patients with MM value and assess meaningful 'benefit-risk' when making treatment decisions, which can be useful for facilitating physician-patient communications and shared decision making.
Fifer, 2020	Journal article	To examine the treatment preferences of people living with MM compared to the treatment preferences of other groups involved in treatment decision making, including carers, as	MM patients, carers, physicians and nurses	Discrete choice experiments. Attributes and levels of the attributes were selected based on previous research, literature review, qualitativ	Overall survival and remission period, side effects, mode and frequency of administration, annual out of	Significant heterogeneity in preferences for treatment attributes. In particular, overall survival, remission period and annual out of pocket cost were the attributes with the most variation. In comparison to people living with MM, carers were less cost-sensitive and more concerned with quality of life (remission period). Physicians and nurses were generally more concerned with overall survival and more cost sensitive than people living with MM. This study

		well as physicians and nurses who treat people living with MM in Australia.		e research and expert opinion.	pocket costs	demonstrated that not all people living with MM valued the same treatment attributes equally. Further, not all groups involved in MM treatment decision making had preference alignment on all treatment attributes. This has important implications for healthcare policy decisions and shared decision making. Results from this study could be used to guide decisions around the value of new MM medicines or the medical plan surrounding the needs of those living with MM, as well as those caring for them.

Appendix 3: Overview of treatment characteristics assessed in previous patient preference studies in Multiple Myeloma

Attribute (group) name[4]	Number of preference studies of 14 studies investigating attribute (group)
Psychosocial status	22
Administration	11
Changed levels of blood cells and ions	11
Progression-free-survival	6
Performance status	6
Safety, side effects & adverse reactions	5
(Bone) pain	4
Neuropathy	4
GI problems	4
Overall survival	3
Symptoms	3
HCP interaction	3
Life expectancy	3
Heart failure	3
(Health-related) QoL	2
Quality of care	2
Remission	2

[4] The attributes investigated in preference studies were grouped

Information needs	2
infections	2
Renal failure	2
Nausea and vomiting	2
Toxicity	2
Clinical/molecular differences	1
Treatment response	1
Efficacy	1
Bone lesions/fractures	1
Clitting disorders	1
Paraproteins	1
Spinal cord compression	1
Appetite change	1
Bleeding	1
Breathlessness	1
Cognitive impairment	1
Cough	1
Disturbed taste	1
Dry mouth	1
Performance status	1
Hair loss	1

Insomnia	1
Swollen limb	1
Syncope	1
Tingling and numbness tremor	1
Weight change	1
Thromboembolic events	1

Appendix 4: favorable and unfavorable effects of Multiple Myeloma treatments assessed by the European Medicines Agency

Data extracted from European Assessment Reports of products approved for treatment of Multiple Myeloma from https://www.ema.europa.eu/en/medicines

	Trade name	Active substance	Marketing Authorization status	Benefit/favourable effects (effect measure and size)	Risks/unfavourable effects
1	Ninlaro	Ixazomib	conditional approval, additional monitoring	PFS increase: 6 months higher versus placebo	diarrhoea, constipation, thrombocytopenia, neutropenia, peripheral neuropathy, nausea, peripheral oedema, vomiting and nose and throat infection
2	Darzalex	Daratumumab	additional monitoring	Disappearance of or at least a 50% reduction in a protein found in multiple myeloma cells: 29 (no comparator)	infusion-related reactions and tiredness, fever, nausea, diarrhoea, muscle spasm, upper respiratory tract infections, neutropenia, anaemia, thrombocytopenia, peripheral sensory neuropathy
				PFS increase: 78% of patients vs 52% of patients receiving placebo	
3	Bortezomib Hospira	Bortezomib	full approval	Generic medicine, see bortezomib sun	Generic medicine, see bortezomib sun
4	Thalidomide Celgene	Thalidomide	full approval	Survival time: 18,4 months increase versus placebo	neutropenia,leucopenia,anaemia, lymphopenia, thrombocytopenia, peripheral neuropathy, remor, dizziness, paraesthesia, dysaesthesia, somnolence, constipation, peripheral oedema

5	Lenalidomide Accord	Lenalidomide	additional monitoring	Generic medicine, see lenalidomide	Generic medicine, see lenalidomide
6	Imnovid	Pomalidomide	additional monitoring	PFS: 8 weeks higher versus placebo	anaemia, neutropenia, fatigue, thrombocytopenia, pyrexia, peripheral oedema, peripheral neuropathy, pneumonia
7	Revlimid	Lenalidomide	additional monitoring	PFS: 28 weeks higher versus placebo	bronchitis, nasopharyngitis, cough, gastroenteritis, upper respiratory tract infection, tiredness, neutropenia, constipation, diarrhoea, muscle cramps, anaemia, thrombocytopenia, rash, back pain, insomnia, decreased appetite, fever, peripheral oedema, leucopenia, weakness
8	Farydak	Panobinostat	additional monitoring	PFS: 4 months higher versus placebo	diarrhoea, tiredness, nausea, vomiting, thrombocytopenia, anaemia, neutropenia, lymphopenia, weakness, pneumonia, tachycardia, palpitations, irregular heart rhythms (atrial fibrillation, sinus tachycardia)
9	Kyprolis	Carfilzomib	additional monitoring	PFS: 9,3 months higher versus placebo	anaemia (low red blood cell counts), tiredness, nausea, diarrhoea, thrombocytopenia (low blood platelet counts), fever, dyspnoea (difficulty breathing), respiratory tract (airways) infection, cough and neutropenia (low levels of

					neutrophils, a type of white blood cell).
10	Bortezomib Accord	Bortezo mib	full approval	Generic medicine, see bortezomib sun	Generic medicine, see bortezomib sun
11	Neofordex	Dexamet hazone	full approval	No new clinical trial data presented, reference to literature	hyperglycaemia (high blood sugar levels), insomnia (difficulty sleeping), muscle pain and weakness,<br><br>asthenia, tiredness, oedema, weight increase, pneumonia, other infections, psychiatric disorders such<br><br>as depression
12	Caelyx	Doxorub icin	full approval	PFS: 2,8 months higher versus placebo	nausea, palmar-plantar erythrodysaesthesia syndrome, vomiting, stomatitis, rash, asthenia, low blood cell counts, loss of appetite, alopecia, fatigue, diarrhoea, constipation and mucositis
13	Aplidin	Plitidepsi n	refused	PFS: 1 month higher versus placebo, OS: “not sufficiently demonstrated”	severe side effects (not further specified)
14	Mozobil (used to increase collection of stem cells)	Plerixafo r	full approval	Number of patients in whom a target number of stem cells could be collected from the blood within two or four collection days: 38% higher than placebo	diarrhoea, nausea (feeling sick) and reactions at the site of injection

15	Intron A	Interferon alfa-2b	full approval	Overall survival: "effective"	pharyngitis, viral infection, leucopenia, loss of appetite, depression, insomnia, anxiety, emotional lability, agitation, nervousness, dizziness, headache, impaired concentration, dry mouth, blurred vision, dyspnoea, coughing, nausea, vomiting, abdominal pain, diarrhoea, stomatitis, dyspepsia, alopecia, pruritus, dry skin, rash, increased sweating, myalgia, arthralgia, musculoskeletal pain, reactions at the site of the injection including inflammation, fatigue, rigors, pyrexia, flu-like symptoms, asthenia, irritability, chest pain, malaise, and weight loss
16	Bortezomib Sun	Bortezomib	full approval	Generic medicine	Generic medicine
17	Empliciti	Elotuzumab	additional monitoring	PFS: 4,2 months higher versus placebo	infusion reactions (with symptoms such as fever and chills), diarrhoea, shingles, sore throat, cough, pneumonia, colds, low levels of white blood cells and weight loss
				Complete clearance of cancer: 13% more patients versus placebo	
18	Velcade	Bortezomib	full approval	PFS: 2,7 months higher versus placebo	nausea, diarrhoea, constipation, vomiting, fatigue, pyrexia, thrombocytopenia, anaemia, neutropenia,

					peripheral neuropathy, headache, paraesthesia, decreased appetite, dyspnoea, rash, herpes zoster, and muscle pain
				Response to treatment: 70% (no comparator)	
19	Blenrep	Belanta mab mafodoti n	conditional approval	Overall response rate of 32%. Response lasted on average for 11 months.	Keratopathy, thrombocytopenia, pneumonia, fever, infusion reactions.

Appendix 5: overview of (un-)favourable effects of MM treatments assessed by the European Medicines Agency as described in the European Assessment Reports (EPARs)

Benefits/favourable effects	Number of medical products/EPARs
PFS increase	10
Survival increase	1
Overall survival	1
Overall response rate	2
Reduction in protein found	1
Increased number of stem cells collected	1
Clearance of cancer	1
Risks/unfavourable effects	Number of medical products/EPARs
Gastro-entero	25
Weight changes	3
Low levels of blood cells	31
Respiratory/lung	22
Musculoskeletal pain	8
Malaise: weakness, tiredness, sleepiness/loss of appetite	25
Nausea/dizziness	10
Insomnia	3
Neurological/abnormal sensations	8
Edema	5

Skin problems: (viral) infections, rash, ...	13
Headache	2
Mental changes: depression, anxiety, emotional lability, agitation, nervousness, impaired concentration	5
Temperature changes; colds/fever	9
Tachycardia	1
Blurred vision	1
Hand-foot syndrome	1
Alopecia	2
Keropathy	1

Appendix 6: overview of phase 3 Multiple Myeloma clinical trials

EU clinical trial database: Clinicaltrialregister.eu

- Search term: multiple myeloma OR Kahler disease OR myelomatosis OR plasma cell myeloma
- No restrictions on country/age
- Trial status: completed, ongoing, restarted
- Trial phase: phase III
- 2010 onwards

→ 120 results (last search on 8st of May 2019)

Excluded if:

- Medical condition is not for treatment of MM (e.g. staging of MM, mobilization of stem cells, graft vs host disease)
- Non-english title
- Investigating diagnostics (e.g. PET)
- For treatment of MM symptoms and drug-induced side effects (e.g. anaemia, bone pain, infections, nephropathy)
- Pharmacogenomic studies
- Studies assessing bioavailability
- Studies not aiming to measure efficacy (e.g. safety studies)

→ included: 61 clinical trials

EudraCT number	Full title
2012-005283-97	A Randomized, Open-label Phase 3 Study of Carfilzomib, Melphalan, and Prednisone versus Bortezomib, Melphalan, and Prednisone in Transplant ineligible Patients with Newly Diagnosed Multiple Myeloma
2015-002993-19	An Open-Label Treatment Use Protocol for Daratumumab in Subjects with Multiple Myeloma Who Have Received at Least 3 Prior Lines of Therapy (Including a Proteasome Inhibitor and an Immunomodulatory
2011-005103-32	A Multicenter, Randomized, Double-Blind, Placebo Controlled Phase 2 Study of LY2127399 in Combination with Bortezomib and Dexamethasone in Patients with Previously Treated Multiple Myeloma
2009-016871-32	A Randomized Phase III Study Comparing Conventional Dose Treatment Using a Combination of Lenalidomide, Bortezomib and Dexamethasone (RVD) to High-Dose Treatment with Peripheral Stem Cell Transplant in the Initial Management of Myeloma in Patients up to 65 Years of Age (IFM/DFCI 2009)

2014-003239-21	An open-label, multi-center, expanded treatment protocol of oral panobinostat in combination with bortezomib and dexamethasone in patients with relapsed and relapsed and refractory multiple myeloma
2010-019820-30	A Phase 3, Multicenter, Randomized, Open-label Study to Compare the Efficacy and Safety of Pomalidomide in Combination with Low-Dose Dexamethasone versus High-Dose Dexamethasone in Subjects with Refractory or Relapsed and Refractory Multiple Myeloma
2010-023343-16	Open-label, multi-center, single-arm study for the safety and efficacy of pomalidomide (CC-4047) monotherapy for subjects with refractory or relapsed and refractory multiple myeloma: a companion study for clinical trial CC-4047-MM-003
2012-000750-66	Randomized Phase III Trial of Lenalidomide Versus Observation Alone in Patients with Asymptomatic High-Risk Smoldering Multiple Myeloma
2017-001940-37	Randomized, Open Label, Multicenter Study Assessing The Clinical Benefit Of Isatuximab Combined With Carfilzomib (Kyprolis®) And Dexamethasone Versus Carfilzomib With Dexamethasone In Patients With Relapse And/Or Refractory Multiple Myeloma Previously Treated With 1 to 3 Prior Lines
2011-004795-11	A Randomized, Controlled, Phase 3 Study to Evaluate Optimized Retreatment and Prolonged Therapy With Bortezomib (VELCADE) in Patients With Multiple Myeloma in First or Second Relapse
2010-021557-40	A phase III trial comparing bortezomib, cyclophosphamide and dexamethasone versus lenalinomide cyclophosphamide and dexamethasone in patients with multiple myeloma at first relapse
2013-002157-29	Clinical, multicenter, single-arm, with a scheme of treatment with low doses of Bortezomib / Melphalan / Prednisone (Velcade) (MPV) in patients with multiple myeloma (MM) newly diagnosed symptomatic> = 75 years.
2015-005699-21	An Open-Label, Randomized Phase 3 Trial of Combinations of Nivolumab, Pomalidomide and Dexamethasone in Relapsed and Refractory Multiple Myeloma
2014-004781-15	Study of Daratumumab in Combination with Bortezomib (VELCADE), Thalidomide, and Dexamethasone (VTD) in the First Line Treatment of Transplant Eligible Subjects with Newly Diagnosed Multiple Myeloma
2007-004823-39	A PHASE III, RANDOMIZED, OPEN-LABEL, 3-ARM STUDY TO DETERMINE THE EFFICACY AND SAFETY OF LENALIDOMIDE (REVLIMID) PLUS LOW-DOSE DEXAMETHASONE WHEN GIVEN

	UNTIL PROGRESSIVE DISEASE OR FOR 18 FOUR-WEEK CYCLES VERSUS THE COMBINATION OF MELPHALAN, PREDNISONE, AND THALIDOMIDE GIVEN FOR 12 SIX-WEEK CYCLES IN PATIENTS WITH PREVIOUSLY UNTREATED MULTIPLE MYELOMA WHO ARE EITHER 65 YEARS OF AGE OR OLDER OR NOT CANDIDATES FOR STEM CELL TRANSPLANTATION
2012-001888-78	A MULTICENTER, SINGLE-ARM, OPEN-LABEL STUDY WITH POMALIDOMIDE IN COMBINATION WITH LOW DOSE DEXAMETHASONE IN SUBJECTS WITH REFRACTORY OR RELAPSED AND REFRACTORY MULTIPLE MYELOMA
2014-000255-85	Phase 3 Study Comparing Daratumumab, Bortezomib and Dexamethasone (DVd) vs Bortezomib and Dexamethasone (Vd) in Subjects With Relapsed or Refractory Multiple Myeloma
2014-001052-39	The FACTOR Study (Filanesib and Carfilzomib Treatment of Relapsed/Refractory Multiple Myeloma): A Multinational, Randomized, Open-label Phase 3 Study of Filanesib (ARRY-520) + Carfilzomib Versus Single-agent Carfilzomib in Patients With Advanced Multiple Myeloma
2017-000044-18	Induction therapy with bortezomib-melphalan and prednisone (VMP) followed by lenalidomide and dexamethasone (Rd) versus carfilzomib, lenalidomide and dexamethasone (KRd) plus/minus daratumumab, 18 cycles, followed by consolidation and maintenance therapy with lenalidomide and daratumumab: phase III, multicenter, randomized trial for elderly fit newly diagnosed multiple myeloma patients aged between 65 and 80 years
2015-004411-20	A Phase 3, Multicenter, Randomized, Double Blind Study of Bortezomib and Dexamethasone in Combination with Either Venetoclax or Placebo in Subjects with Relapsed or Refractory Multiple Myeloma Who are Sensitive or Naïve to Proteasome Inhibitors
2015-001183-19	Lenalidomide and dexamethasone (Ld) versus Clarithromycin / Lenalidomide [Revlimid®] / Dexamethasone (BiRd) as initial therapy in Multiple Myeloma.
2017-002611-34	A Randomized, Open-label, Multicenter, Multiphase Study of JNJ-63723283, an Anti-PD-1 Monoclonal Antibody, Administered in Combination with Daratumumab, Compared with Daratumumab Alone in Subjects with Relapsed or Refractory Multiple Myeloma.
2013-005525-23	Phase 3 Study Comparing Daratumumab, Lenalidomide, and Dexamethasone (DRd) vs Lenalidomide and Dexamethasone (Rd) in Subjects With Relapsed or Refractory Multiple Myeloma

2016-003097-41	A Phase 3 Randomized, Open-label, Multicenter Study Comparing Isatuximab (SAR650984) in Combination with Pomalidomide and Low-dose Dexamethasone versus Pomalidomide and Low-dose Dexamethasone in Patients with Refractory or Relapsed and Refractory Multiple Myeloma
2012-000128-16	A Randomized, Open-label, Phase 3 Study of Carfilzomib Plus Dexamethasone vs Bortezomib Plus Dexamethasone in Patients With Relapsed Multiple Myeloma
2014-002273-11	A Phase 3 Study Comparing Daratumumab, Lenalidomide, and Dexamethasone (DRd) vs Lenalidomide and Dexamethasone (Rd) in Subjects with Previously Untreated Multiple Myeloma who are Ineligible for High Dose Therapy
2014-002272-88	A Phase 3, Randomized, Controlled, Open-label Study of VELCADE (Bortezomib) Melphalan-Prednisone (VMP) Compared to Daratumumab in Combination with VMP (D-VMP), in Subjects with Previously Untreated Multiple Myeloma who are Ineligible for High-dose Therapy
2017-002238-21	A Phase 3 Randomized, Open-label, Multicenter Study Assessing the Clinical Benefit of Isatuximab (SAR650984) in Combination with Bortezomib (Velcade®), Lenalidomide (Revlimid®) and Dexamethasone versus Bortezomib, Lenalidomide and Dexamethasone in Patients with Newly Diagnosed Multiple Myeloma (NDMM) Not Eligible for Transplant
2017-000206-38	A Phase 3 Randomized, Multicenter Study of Subcutaneous vs. Intravenous Administration of Daratumumab in Subjects With Relapsed or Refractory Multiple Myeloma
2016-003554-33	A Randomized, Open-label, Phase 3 Study Comparing Carfilzomib, Dexamethasone, and Daratumumab to Carfilzomib and Dexamethasone for the treatment of Patients With Relapsed or Refractory Multiple Myeloma
2010-020347-12	A Phase 3, Randomized, Open Label Trial of Lenalidomide/dexamethasone With or Without Elotuzumab in Relapsed or Refractory Multiple Myeloma
2013-001729-26	Phase 3b, Randomized Trial of Revlimid® (Lenalidomide) Versus Placebo Maintenance Therapy Following Melphalan Prednisone Velcade® (Bortezomib) Induction Therapy in Newly Diagnosed Multiple Myeloma
2010-022445-20	A Phase 3, Randomized, Open Label Trial of Lenalidomide/dexamethasone With or Without Elotuzumab in Subjects with Previously Untreated Multiple Myeloma.
2017-001618-27	A Phase 3 Study Comparing Pomalidomide and Dexamethasone With or Without Daratumumab in Subjects With Relapsed or Refractory Multiple

	Myeloma Who Have Received at Least One Prior Line of Therapy With Both Lenalidomide and a Proteasome Inhibitor.
2011-005496-17	A Phase 3, Randomized, Double-Blind, Multicenter Study Comparing Oral MLN9708 Plus Lenalidomide and Dexamethasone Versus Placebo Plus Lenalidomide and Dexamethasone in Adult Patients With Relapsed and/or Refractory Multiple Myeloma
2015-002901-12	A phase III study of Lenalidomide and low-dose Dexamethasone with or without Pembrolizumab (MK3475) in newly diagnosed and treatment naïve Multiple Myeloma (KEYNOTE 185).
2014-001394-13	A Phase 3, Randomized, Placebo-Controlled, Double-Blind Study of Oral Ixazomib<br><br>Maintenance Therapy After Initial Therapy in Patients With Newly Diagnosed Multiple Myeloma Not Treated With Stem Cell Transplantation
2015-002509-13	A phase III study of Pomalidomide and low dose Dexamethasone with or without Pembrolizumab (MK3475) in refractory or relapsed and refractory Multiple Myeloma (rrMM) (KEYNOTE 183)
2014-000268-17	A PHASE 3, MULTICENTER, RANDOMIZED, OPEN-LABEL STUDY TO COMPARE THE EFFICACY AND SAFETY OF POMALIDOMIDE, BORTEZOMIB AND LOW-DOSE DEXAMETHASONE VERSUS BORTEZOMIB AND LOW-DOSE DEXAMETHASONE IN SUBJECTS WITH RELAPSED OR REFRACTORY MULTIPLE MYELOMA
2013-002076-41	A Phase 3, Randomized, Placebo-Controlled, Double-Blind Study of Oral Ixazomib Citrate (MLN9708) Maintenance Therapy in Patients With Multiple Myeloma Following Autologous Stem Cell Transplant
2015-002509-13	A phase III study of Pomalidomide and low dose Dexamethasone with or without Pembrolizumab (MK3475) in refractory or relapsed and refractory Multiple Myeloma (rrMM) (KEYNOTE 183)
2014-000268-17	A PHASE 3, MULTICENTER, RANDOMIZED, OPEN-LABEL STUDY TO COMPARE THE EFFICACY AND SAFETY OF POMALIDOMIDE, BORTEZOMIB AND LOW-DOSE DEXAMETHASONE VERSUS BORTEZOMIB AND LOW-DOSE DEXAMETHASONE IN SUBJECTS WITH RELAPSED OR REFRACTORY MULTIPLE MYELOMA
2013-002076-41	A Phase 3, Randomized, Placebo-Controlled, Double-Blind Study of Oral Ixazomib Citrate (MLN9708) Maintenance Therapy in Patients With Multiple Myeloma Following Autologous Stem Cell Transplant

2008-008606-52	A PHASE 3, INTERGROUP MULTICENTRE, RANDOMIZED, CONTROLLED 3 ARM PARALLEL GROUP STUDY TO DETERMINE THE EFFICACY AND SAFETY OF LENALIDOMIDE IN COMBINATION WITH DEXAMETHASONE (Rd) VERSUS MELPHALAN, PREDNISONE AND LENALIDOMIDE (MPR) versus CYCLOPHOSPHAMIDE, PREDNISONE AND LENALIDOMIDE (CPR) IN NEWLY DIAGNOSED MULTIPLE MYELOMA SUBJECTS
2016-003517-95	A Randomized, Controlled, Open-Label, Phase 3 Study of Melflufen/ Dexamethasone Compared with Pomalidomide/Dexamethasone for Patients with Relapsed Refractory Multiple Myeloma who are Refractory to Lenalidomide
2016-001205-16	A Phase 3 Randomized, Multicenter Study of Subcutaneous Daratumumab Versus Active Monitoring in Subjects with High-risk Smoldering Multiple Myeloma
2016-004742-28	A Phase 2/3, Randomized, Open-Label Study Comparing Oral Ixazomib/Dexamethasone and Oral Pomalidomide/Dexamethasone in Relapsed and/or Refractory Multiple Myeloma
2018-002992-16	A Phase 3 Study Comparing Daratumumab, VELCADE (bortezomib), Lenalidomide, and Dexamethasone (D-VRd) vs VELCADE, Lenalidomide, and Dexamethasone (VRd) in Subjects with Previously Untreated Multiple Myeloma who are Eligible for High-dose Therapy
2018-001545-13	A Phase 3 Study Comparing Daratumumab, VELCADE (bortezomib), Lenalidomide, and Dexamethasone (D-VRd) with VELCADE, Lenalidomide, and Dexamethasone (VRd) in Subjects with Untreated Multiple Myeloma and for Whom Hematopoietic Stem Cell Transplant is Not Planned as Initial Therapy
2016-003957-14	A Phase 3 Randomized, Controlled, Open-label Study of Selinexor, Bortezomib, and Dexamethasone (SVd) versus Bortezomib and Dexamethasone (Vd) in Patients with Relapsed or Refractory Multiple Myeloma (RRMM)
2013-000326-54	A Phase 3, Randomized, Double-Blind, Multicenter Study Comparing Oral MLN9708 Plus Lenalidomide and Dexamethasone Versus Placebo Plus Lenalidomide and Dexamethasone in Adult Patients With Newly Diagnosed Multiple Myeloma
2015-002380-42	Phase 3 Randomized trial of carfilzomib, lenalidomide, dexamethasone versus lenalidomide alone after stem-cell transplant for multiple myeloma.

2014-000634-34	IFM 2014-02 study: A randomized phase III study of Bortezomib-Melphalan 200 conditioning regimen versus Melphalan 200 for frontline transplant eligible patients with multiple myeloma
2005-002756-18	Bortezomib consolidation in patients with myeloma following treatment with high-dose melphalan and autologous stem cell support. A randomised NMSG trial (15/05)
2017-001616-11	Elotuzumab (E) in Combination with Carfilzomib, Lenalidomide and Dexamethasone (E-KRd) versus KRd prior to and following Autologous Stem Cell Transplant in Newly Diagnosed Multiple Myeloma and Subsequent Maintenance with Elotuzumab and Lenalidomide versus Single-Agent Lenalidomide<br><br>A phase III study by DSMM (Deutsche Studiengruppe Multiples Myelom)
2009-016616-21	Lenalidomide, Adriamycin, Dexamethasone (RAD)<br><br>Versus Lenalidomide, Bortezomib, Dexamethasone (VRD) for Induction in Newly Diagnosed Multiple Myeloma followed by Response-adapted Consolidation and Lenalidomide Maintenance - A Randomized Multicenter Phase III Trial by Deutsche Studiengruppe Multiples Myelom (DSMM XIV)
2018-000665-36	A Randomized, Open-label, Phase 3 Study Comparing Once-weekly vs Twice-weekly Carfilzomib in Combination with Lenalidomide and Dexamethasone in Subjects With Relapsed or Refractory Multiple Myeloma (A.R.R.O.W.2)
2014-000554-10	A randomized, open-label, national multicenter, phase III trial studying maintenance treatment with lenalidomide and dexamethasone versus lenalidomide, dexamethasone and MLN9708 after autologous hematopoietic stem cell transplant in patients with newly-diagnosed, symptomatic multiple myeloma.
2008-008599-15	A PHASE 3, MULTICENTRE, RANDOMIZED, CONTROLLED STUDY TO DETERMINE THE EFFICACY AND SAFETY OF CYCLOPHOSPHAMIDE, LENALIDOMIDE AND DEXAMETHASONE (CRD) versus MELPHALAN (200 mg/m2) FOLLOWED BY STEM CELL TRANSPLANT IN NEWLY DIAGNOSED MULTIPLE MYELOMA SUBJECTS
2017-004768-37	A randomized phase III trial assessing the benefit of the addition of isatuximab to lenalidomide / bortezomib / dexamethasone (RVd) induction and lenalidomide maintenance in patients with newly diagnosed multiple myeloma
2013-003174-27	A PHASE III STUDY OF VELCADE (BORTEZOMIB) THALIDOMIDE DEXAMETHASONE (VTD) VERSUS VELCADE (BORTEZOMIB) CYCLOPHOSPHAMIDE DEXAMETHASONE (VCD) AS AN INDUCTION TREATMENT PRIOR TO AUTOLOGOUS STEM CELL

	TRANSPLANTATION IN PATIENTS WITH NEWLY DIAGNOSED MULTIPLE MYELOMA.

Appendix 7: overview of endpoints and adverse events reported in Multiple Myeloma phase 3 clinical trials

**I. Primary and secondary endpoints**
1. Survival
Progression-free-survival (PFS)
PFS with a later cut-off date
PFS2
Overall survival (OS)
OS with later cut-off date
OS based on final dataset
2. Response to treatment
Response rate (RR)
Overall response rate (ORR)
Partial response rate (PRR)
complete response rate (CRR)
Disease control rate (DCR)
Time to recurrence (TTR)
Best Overall Response (BOR)
Very good partial response (VGPR)
stringent complete response (sCR)

Percentage of Participants with an Objective Response
Time to Response
Quality of Response [QoR]
Time to progression (TTP)
Duration of Response (DoR)
Time to First Skeletal-Related Event (SRE)
Time to Next Treatment/therapy (TNT)
Time to first-line treatment for MM
Time to the First Hemoglobin Improvement
Measurement of stable disease (SD)
Time to need for relapse treatment
3. Changes in MM treatment adverse events or MM symptoms
percentage of Participants With ≥ Grade 2 Peripheral Neuropathy
Number of Participants with >30% Reduction in Brief Pain Inventory (BPI)
Number of Participants With Adverse Events (AEs) and Type of Adverse Events
Worst Pain Score
Pain response rate
Change in pain severity/interference
Time to Improvement in Bone Pain
Time to Improvement in Renal Function

Time to Improvement in Performance Status
Improvement in CRAB criteria (renal function, haematological function, infection rate)
Biochemical or diagnostic progression (SLiM-CRAB)
Percentage of Participants with a Significant Reduction in Left Ventricular Ejection Fraction (LVEF)
Change from Baseline in Right Ventricular Fractional Area Change (FAC)
Change from Baseline in Pulmonary Artery Systolic Pressure (PASP)
4. Quality of life
EORTC Quality of Life QLQ-C30 and QOL Scores
EORTC QLQ-MY20
Euro Quality of Life (EQ-5D-5L)
Health utility
Cost utility
Health related QoL
5. Other
Minimal residual disease (MRD)
Clinical benefit rate (ORR+minimal response),
Patient reported outcomes
EFS
Relative dose intensity

Convenience as measured by the Patient-reported Convenience With Carfilzomib-dosing Schedule Question after cycle 4 of treatment
**II. Adverse events**
1. Respiratory, thoracic and mediastinal disorders
Cough
2. Blood and lymphatic system disorders
Deep vein thrombosis
Haemorrhage
Hypertension
Hypotension
Hypovolaemic shock
orthostatic hypotension
shock haemorrhagic
Anaemia
Leukopenia
Thrombocytopenia
Neutropenia
Pancytopenia
Hyperviscosity syndrome
3. Malignancies
Benign Neoplasm Of Bladder

Multiple Myeloma
Prostate Cancer
Basal cell carcinoma,
Plasma cell leukaemia,
Plasmacytoma,
acute myeloid leukaemia
colorectal cancer
hepatic neoplasm
plasma cell leukaemia
plasma cell myeloma
4. Nervous system disorders
altered state/loss of consciousness
Dizziness
Headache
Neuralgia
Ischaemic stroke
autonomic neuropathy
Neuropathy peripheral
Peripheral sensory neuropathy
Paraesthesia

Spinal Cord Compression
5. General disorders and administration site conditions
General physical health deterioration
Disease progression
General physical health deterioration
Localised oedema
Multi-organ failure
Multiple organ dysfunction syndrome
Oedema peripheral
Light headed
Confusional state
Asthenia
Fatigue
Pyrexia
Insomnia
Chills
6. Clinical measurements
Blood creatinine increased
decreased neutrophil count
decreased white blood cells

platelet count decreased
renal function test abnormal
troponin increased
hypokalemia
hypocalcaemia
alanine aminotransferase increased
Hyperglycaemic Hyperosmolar Nonketotic Syndrome
Hypomagnesaemia
Hyperproteinaemia
weight decreased
Immunogenicity/Antibodies against treatment
7. Cardiac disorders
sinus tachycardia
acute coronary syndrome
acute myocardial infarction
atrial fibrillation
cardiac failure (congestive)
angina pectoris
left ventricle outflow tract obstruction
cardiac arrest

ventricular dysfunction
8. Pulmonary disorders
Pulmonary embolism
Pulmonary oedema
Respiratory failure
acute respiratory failure
chronic obstructive pulmonary disease
dyspnoea
interstitial lung disease
pleural effusion
pneumonitis
9. Gastro interstinal disorders
Dysphagia
Colitis
Gastric perforation
Ileus paralytic
intestinal ischaemia
large intestinal haemorrhage
melaena
nausea

(upper) gastrointestinal haemorrhage
haematuria
incontinence
renal failure (acute)
cholecystitis (acute)
Constipation
Diarrhoea
Nausea
Vomiting
abdominal distension
abdominal pain
dyspepsia
Acute kidney injury,
Intestinal infarction
10. Skin and subcutaneous tissue disorders
Rash
11. Musculoskeletal and connective tissue disorders
Back pain
Bone pain
Muscular weakness

Muskuloskeletal (chest) pain
Pathollogical fracture
Pain in extremity
Osteolyse
Arthralgia
Muscle spasms
12. Metabolism and nutrition disorders
cachexia
Decreased appetite
Hypokalaemia
Dehydratation
diabetes mellitus
Diabetic ketoacidosis
hyperglycaemia
hypovolaemia
Hypercalcaemia
Hyponatraemia
13. Infections and infestations
Upper respiratory tract infection
lower respiratory tract infection

Bacteraemia
Brain abscess
bronchopneumonia
cellulitis
cytomegalovirus colitis
diverticulitis
helicobacter infection
herpes zoster
infection,
infectious colitis
Influenza/influenza like illness
lobar pneumonia,
otitis media,
pneumococcal sepsis
pneumocystis jiroveci pneumonia
pneumonia (haemophilus)
respiratory syncytial virus infection
(upper/lower) respiratory tract infection
(Neutropenic) sepsis
sepsis

septic shock
sinusitis
soft tissue infection
urinary tract infection
wound infection
lung infection/pulmonary infection
Bronchitis
Device related infection
Nasopharyngitis
Clostridium Difficile Colitis
Enterocolitis Infectious
Erysipelas
Gastroenteritis
Infection
Meningitis Cryptococcal,
Otitis Media
Subcutaneous Abscess
14. Other
Number of hospital stays and hospitalisation days

Appendix 8: discussion guides used for the nominal group technique (NGT)

I. Before the discussion

1. Roles and responsibilities of the moderator and assistant

- The moderator speaks the native language of participants
- This guide, the information sheets, informed consents, answer sheets (Appendix 1) and PowerPoint slides supporting the discussion should be available in the native language of participants, prior to the discussion
- The information sheets, informed consents and answer sheets should be provided to participants before the discussion
- The answer sheets (Appendix 1) should be completed by participants and returned to the moderator
- The averages for each of the characteristics should be calculated using the completed answer sheets (section 3 of Appendix 1)
- In order to prepare for the focus group, a meeting between moderator and assistant should be planned before the focus group to go over this protocol and make sure the tasks are understood by the moderator and assistant

a. *Moderator*

- Takes care of the organizational tasks listed below
- Exercises mild unobtrusive control; moderates the discussion
- Has adequate knowledge of topic
- Appears like the participants: not formal (e.g. no suits, no blazers)
- Uses purposeful small talk in the beginning to make participants feel comfortable
- Is alert and free from distractions: puts sound of phone off and puts phone away during discussion
- Has the discipline of listening and applies active listening:
  - Uses verbal reactions:
    - Short verbal responses (e.g. “I see”, “Yes”, “Okay”, avoid "that's good", "excellent")
    - Pauses and probes and 5 second pause probes (e.g. "Would you explain further?" "Would you give an example?")
    - Listens for inconsistent/vague/cryptic comments and probes for understanding (e.g. "I don't understand")
    - Considers asking a final yes/no question
    - Uses probes to refocus the discussion when the discussion goes off-topic (e.g. “Now that we have talked about XXX, I would go back/address the question/topic…”)
  - Uses nonverbal reactions:
    - Head nodding
- Knows this protocol including the questions very well
- Takes into account the different types of participants and tries to balance the conversation while addressing the obligatory topics and questions: dominant talkers, shy participants, etc.

b. *Assistant*
- Handles logistics (e.g. PowerPoint slides, audio recording); monitors audio recording equipment and PowerPoint slide show; puts up correct slide at correct time
- Appears like the participants: not formal (e.g. no suits, no blazers)
- Takes careful notes on paper or on laptop:
    - Anticipates that others will use these notes. Notes sometimes are interpreted days or weeks following the focus group when memory has faded. Consistency and clarity are essential
    - It is essential that this information is easily identified and organized. The notes should be divided according to the focus group questions/topics and include time indications
    - The notes will contain different types of information:
        - Quotes:
            - Listen for notable quotes, the well said statements that illustrate an important point of view. Listen for sentences or phrases that are particularly enlightening or eloquently express a particular point of view. Place initials of speaker after the quotations. Usually, it is impossible to capture the entire quote. Capture as much as you can with attention to the key phrases. Use three periods ... to indicate that part of the quote was missing.
        - Key points and themes for each question:
            - Typically, participants will talk about several key points in response to each question. These points are often identified by several different participants. Sometimes they are said only once but in a manner that deserves attention. In the summary at the end of the focus group the assistant moderator will share these themes with participants for confirmation.
        - Follow-up questions that could be asked:
            - Sometimes the moderator may not follow-up on an important point or seek an example of a vague but critical point. The assistant moderator may wish to follow-up with these questions at the end of the focus group
        - Big ideas, hunches, or thoughts:
            - Occasionally the assistant will discover a new concept; the assistant may suddenly understand something she/he before did not, or he/she may find an important theme. These insights should be included in the summary as they are helpful in later analysis
        - Other factors:
            - Make note of factors which might aid analysis such as passionate comments, body language, or non-verbal activity. Watch for head nods, physical excitement, eye contact between certain participants, or other clues that would indicate level of agreement, support, or interest

- These notes will be used by the assistant at the end of the focus group discussion to summarize the focus group (see end protocol)

- Controls for equal participation by all participants and informs the moderator if some participants are not getting the chance to participate
- Knows this protocol and especially the tasks for the assistant very well
- Manages time via discrete signs to the moderator
- Gives a general summary based upon the abovementioned notes at the end of the focus group. The summary should encompass the different questions/topics addressed in the discussion and give a balanced view of the different opinions expressed
- *General tips for the discussion:*
  - If one participant tries to dominate the session, the moderator should invite each person to speak in turn. Build some predefined chat prompts to help diffuse or deflect the discussion.
  - Avoid personal confrontation and allow the group to police itself (e.g. “do others in the group agree?”) . Make sure you have defined the group rules before the group discussion begins. This can be done by presenting a page to the group with a basic list of rules or guidelines to follow.
  - Participants will have many different attitudes and prejudices. Don’t ignore these – work with them. Just say: “We seem to have a difference of opinion here. Let’s talk about it together. Why do we all think differently on this topic?” Then let the group discuss it. Use differences of opinion as a topic of discussion. As a moderator you should always avoid taking sides!
  - If the group is slow off the mark promote and foster discussion. Ask open-ended questions. Ones that cannot be answered with just a YES or a NO but rather begin with: How? What? Why?
  - From the beginning, adopt a `listening’ rather than a `questioning’ approach. Start with general issues and use your moderator control panel to make sure you cover the issues you need to cover. Questions do not need to be followed in the order they are presented in the time-line. If the discussion naturally leads to a different topic follow this, but make sure everything is eventually covered.
  - If things get heated during the session, it may be wise to ‘park’ the issue and address it later, either as a private chat or as part of a separate group discussion
  - Dealing with technical issues (especially if the discussion is held online). Despite planning and practice your group members may still run into trouble. If a participant gets disconnected make sure they know they can log right back in and rejoin the group at any time. Keep a phone number or email address of the participant nearby in case you need someone to follow-up to make sure the participant is able to continue.

2. Organizational tasks (moderator)

- Makes sure between 5-7 participants per focus group are recruited, using the following inclusion criteria: i) MM patients diagnosed with symptomatic MM; ii) understanding the used language in the discussion and iii) ability to participate in the discussion.
- Arranges the discussion
- Prepares the slide show for the focus group. This presentation needs to include:
  - A general introduction to the study and its aim

    - The questions
    - The characteristics as found in literature with explanations
    - A slide that will show the average grades for each of the characteristics
- Makes sure the participants receive instructions to enter the discussion

II. <u>The day of the discussion</u>

1. Organizational aspects to be taken care of by the moderator

Prepare the online discussion:

- Ensure the slides are visible
- Ensure audio recorders are there

2. Focus group agenda

Italic is to be said to participants. In **bold** an indication of timing of actions is given. Normal text indicates words that should not be spoken out loud, these are the tasks that should be performed by the moderator and/or assistant.

00:00 Welcome the participants while they arrive

- Create warm and friendly environment
    - Interact with participants and stimulate interaction between them

00:10 Check whether all participants have arrived. If not, the assistant will try to reach these persons via. If the missing participants cannot be reached, the focus group will start without these persons. Ensure that all participants have signed their informed consent form.

Start the focus group with a general introduction:

- *Welcome, my name is* (your first name) *and I will be your moderator today. In addition, I brought* (first name of assistant) *to help me with the focus group. My role as moderator will be to guide the discussion*
- *First of all, has anyone been already involved in a group discussion, also known as a focus group, for research previously?*
    - If so: *this focus group may be different to your previous experience as this is a more structured focus group with 4 different stages.*
- *The purpose of a focus group is to generate ideas at a group level, rather than personal detailed experiences. As I stated earlier, today's group is going to be a structured 4 stage process so keeping to time will be really important. So please forgive us if we have to cut anything short to stay on time, we will be happy to continue further discussions on an individual basis after the formal group session has ended. We should not take longer than 2 hours.*

- *Before this discussion, we asked that you completed an answer sheet. We will go through each of the questions mentioned in the answer sheet and discuss them today. Make sure you have your answer sheet in front of you.*
- *Now I will briefly introduce todays' topic and task. Today, several drugs are being developed for the treatment of multiple myeloma. These drugs have different types and amounts of positive and negative effects. Decision-makers such as pharmaceutical companies, regulators and payers are responsible for deciding what drugs will become available to you as patients. For these decisions, they need to make up their minds of how important these effects are to patients. Therefore, we want to understand how important you, as the eventual end-users of these treatments, find these treatments, via this focus group. Today we will be discussing your thoughts and opinions on what key treatment characteristics matter to you when thinking about treatments for multiple myeloma. The purpose of this discussion is to answer the following question: "What do you think are the most important features of multiple myeloma treatment?"*
- *This study is part of a large European project called PREFER. This research project looks at how and when patients' preferences for new treatments should be incorporated into the drug development process. The opinions collected today will not be used to change anything about your current or future treatment.*
- *The opinions collected today will be used to develop a survey that we will spread out to a large amount of multiple myeloma patients. This will allow us to put in numbers the opinions we collect today*
- *This study is part of a large European project called PREFER. This research project looks at how and when patient preferences for new treatments should be incorporated into the drug development process*

00:13 Explain the "rules":

- *There are no right or wrong answers, only differing points of view*
- *It is possible that you do not agree with all opinions, but please listen respectfully to each other*
- *Since this is an informal discussion, we will address each other only by their first name as indicated on the name cards*
- *We ask you to turn off the sound of your phones*
- *If there are any questions or terms that are used during the focus group that are not clear to you, please let us know*
- *To be able to fully focus on the focus group as moderator and assistant, we will audio record our conversation today. If you do not want that your opinions are being audio recorded, you should not sign the information sheet nor choose to participate in the focus group*
- *To be able to fully understand what everybody says and also to help our analysis, it would be very helpful for the analysis if only one person is speaking at a time*
- *There will be a break in the middle of the discussion of 10 minutes*
- *Are there any questions about what I just mentioned to you?*
- *We will now start the recording. Is that OK for everybody?*

00:14 *Before we start and to get to know each other, we would like to do a round-the-table where each of us introduces themselves in one minute maximally, by stating their name and why you decided to join today's discussion.*

PHASE 1: IDEA

00:20 *As I mentioned earlier, today we want to identify those characteristics of a multiple treatment that you, both individually and as a group, consider to be important. Remember the car example I talked about a moment ago, there are no right or wrong answers, it is about what is important to you, as patients. I want you to turn to section 2 of the answer sheet, entitled 'Identifying treatment characteristics that matter most to you'. We asked you to complete the following questions indivually:*

1. *When you undergo a treatment for multiple myeloma, what* ***improvement*** *do you expect from it? With improvement we mean benefits, favorable or desirable effects. Please also explain why*
2. *Multiple myeloma treatments may also be associated with side-effects. With side-effect we mean risks or undesirable effects of the treatment. Imagine you would start a certain treatment, what* ***side-effects*** *would make you want to doubt whether you want to start taking it? Please also explain why*
3. *Imagine you have started taking a certain treatment, what* ***improvements*** *would make you want to accept more of the side-effects you listed? Please also explain why*
4. *Imagine you have started taking a certain treatment, what* ***side-effects*** *would make you want to reconsider whether you want to continue the treatment? Please also explain why*
5. *Are there any* ***other treatment characteristics****, besides the side-effects and improvements, that* ***would influence your choice*** *to start, continue or stop taking a multiple myeloma treatment? Please also explain why*

PHASE 2: ROUND ROBIN

00:30 *Here we have the characteristics written on the slide that you have noted down. Now we are going to go around the room and one-by-one each state one characteristic that you have listed. We will go around until we run out of characteristics. If someone else states a characteristic that you have written, you don't need to say it again. Please do not forget to mention why you considered the aspects you wrote down as important; and remember that there are no wrong answers, each response is very valuable to us.*

0:45 *Now I would like you to look at the part of the answer sheet where it is mentioned: 'List of treatment characteristics and explanations'. On it are characteristics that we identified in literature. These characteristics are both positive and negative effects related to multiple myeloma or multiple myeloma treatments that are currently being developed by pharmaceutical industry or of treatments already on the market. You may notice similarity to the list you, as a group, have just compiled. It is important to note that the chance these effects may occur in a specific patient may be very different for each of the characteristics; some negative effects listed occur vary rarely and others more frequently. The chances these effects occur and depend on different factors (such as the current treatment, disease history, patient characteristics,…). Therefore, the chance these effects may occur may differ from patient to patient.*

The assistant puts the treatment characteristics on the slides.

PHASE 3: CLARIFICATION AND FINALIZATION

0:55 *We want to make sure that we all have the same understanding for each of the listed characteristics. We will go over each of them and explain them to you*

The moderator goes through all these characteristics of the list and explains them using the explanations provided

1:05 Discussion on the list and integrating list with characteristics of the first part of the focus group:

- *What do you think about this list?*
- *Are there any characteristics you see in this list that were not written down by any of you individually?*
    - If there are characteristics missing from the slide, the assistant will add them and ask participants to add them to the list
    - If there are characteristics on the slide that are not important to participants, the assistant will take them from the list and ask participants to scratch them from the list
- Finalizing the list:
    - The assistant will put up slide with final list and explanations. If there is no explanation available for a certain characteristic, the group will discuss and come up with an explanation

01:15 Break. *We will now have a break of 10 minutes. Please hand in your grading sheet to the assistant. Feel free to use the restroom, take water/coffee/tea/something to eat.*

PHASE 4: GRADING

During the break, ***average*** grades are put on a slide but waits with showing it on the screen.

01:25 *Now we want to do another round of the table where each participant states their most and least important characteristic and why they gave it a high or low score (1 min per person).*

01:50 Assistant puts up slide that gives average scores per characteristic.

Here on the slide you can see the total score of the group. A small score means that the characteristic is not important to all of you. A big score means that it is very important.

- *What do you think about these scores?*

Now we want to use these scores to reach agreement on which of them you find most important.

The group discusses and tries to reach consensus on the 10 most important effects.

- *Are there effects you find more or less important among these 10?*
- *Are there effects that you wrote down that should be added to this list?*
- *Do you agree with this final list?*

02:00 Hand in grading forms**.** *The focus group is now finished*

- Summarize the lessons learnt of today's discussion – by assistant

- Ask if the summary is correct, or if you have forgotten something
- Ask if there are any questions
- You have the opportunity to talk to your treating physician (shortly) after this focus group about your therapy if you wish. Please let us know if you would like to make use of this opportunity; we will pass on this information to your treating physician after this discussion
- Thank all participants for their participation

Collect the following materials and put them on SharePoint:

- Consent forms
- Answer sheets
- Recordings
- Notes

Appendix 9: Participants' characteristics across the four countries

PIs= Proteasome inhibitors; IMiDs= immunomodulating agents; mAbs= monoclonal antibodies

* Statistical differences were identified between the patient groups of the different countries for the following patient characteristics: activity level (X^2=10.78, p=.03), living situation (X^2=4.70, p=.04), enrolment in clinical trial (X^2=8.33, p=.04), years since diagnosis (F=3.28, p=.04).

	Belgium (n= 6)	Finland (n= 6)	Romania (n= 6)	Spain (n= 6)	Total (n= 24)
Age					
M (sd) - range	63 (6) - 18	65 (10) - 26	57 (8)- 24	60 (9) - 19	61 (9) - 27
Gender					
Males: n (%)	3 (50%)	2 (33%)	2 (33%)	4 (67%)	46 (50%)
Females: n (%)	3 (50%)	4 (67%)	4 (67%)	2 (33%)	54 (50%)
Education					
Primary education	0 (0%)	0 (0%)	0 (0%)	1 (17%)	1 (4%)
Secondary education (high school)	1 (17%)	1 (17%)	2 (33%)	2 (33%)	6 (25%)
Bachelor degree (college or university)	1 (17%)	2 (33%)	3 (50%)	0 (0%)	6 (25%)
Master degree (university)	3 (50%)	3 (50%)	1 (17%)	3 (50%)	10 (42%)
Missing answers	1 (17%)	0 (0%)	0 (0%)	0 (0%)	1 (4%)

Activity level*					
No limitations	1 (17%)	4 (67%)	1 (17%)	0 (0%)	6 (25%)
Fair mobility	4 (67%)	1 (17%)	3 (50%)	6 (100%)	14 (58%)
Sedentary	1 (17%)	0 (0%)	2 (33%)	0 (0%)	3 (13%)
Missing answers	0 (0%)	1 (17%)	0 (0%)	0 (0%)	1 (4%)
Living situation*					
Not alone	6 (100%)	3 (50%)	6 (100%)	6 (100%)	21 (88%)
Alone	0 (0%)	2 (33%)	0 (0%)	0 (0%)	2 (8%)
Missing answers	0 (0%)	1 (17%)	0 (0%)	0 (0%)	1 (4%)
Work status					
Employed	2 (33%)	1 (17%)	6 (100%)	3 (50%)	12 (50%)
Retired	3 (50%)	4 (67%)	0 (0%)	3 (50%)	10 (42%)
Other	1 (17%)	0 (0%)	0 (0%)	0 (0%)	1 (4%)
Missing answers	0 (0%)	1 (17%)	0 (0%)	0 (0%)	1 (4%)
Years since diagnosis*					
M (sd) - range	9 (6) - 16	6 (4) - 8	4 (3)- 8	1 (1) - 2	5 (5) - 18
Missing answers	0 (0%)	0 (0%)	0 (0%)	1 (17%)	1 (4%)
Current MM treatment					

Chemotherapy	0 (0%)	1 (17%)	0 (0%)	0 (0%)	1 (4%)
IMiDs	4 (67%)	3 (50%)	1 (17%)	3 (50%)	11 (46%)
mAbs	0 (0%)	2 (33%)	3 (50%)	0 (0%)	5 (21%)
PIs	1 (17%)	3 (50%)	6 (100%)	1 (17%)	12 (50%)
Steroids	1 (17%)	4 (67%)	5 (83%)	1 (17%)	11 (46%)
Bisphosphonates	0 (0%)	0 (0%)	0 (0%)	2 (33%)	2 (8%)
Supportive treatment	2 (33%)	2 (33%)	1 (17%)	3 (50%)	8 (33%)
No current treatment	1 (17%)	0 (0%)	0 (0%)	0 (0%)	1 (4%)
Number of treatment lines					
M (sd) - range	2 (1) - 2	4 (2) - 3	4 (2)- 4	2 (1) - 2	3 (2) - 6
Missing answers	1 (17%)	3 (50%)	0 (0%)	3 (50%)	7 (29%)
Current or previous enrolment in clinical trial*					
Yes: n (%)	2 (33%)	5 (83%)	2 (33%)	1 (17%)	10 (42%)
No: n (%)	4 (67%)	0 (0%)	4 (67%)	5 (83%)	13 (54%)
Missing answers	0 (0%)	1 (17%)	0 (0%)	0 (0%)	1 (4%)

Frequent contact with patient organisation					
Yes: n (%)	2 (33%)	0 (0%)	0 (0%)	0 (0%)	2 (8%)
No: n (%)	4 (67%)	5 (83%)	5 (83%)	6 (100%)	20 (83%)
Missing answers	0 (0%)	1 (17%)	1 (17%)	0 (0%)	2 (8%)
Other chronic health problems					
Yes: n (%)	4 (67%)	3 (50%)	0 (0%)	2 (33%)	9 (38%)
No: n (%)	2 (33%)	2 (33%)	6 (100%)	4 (67%)	14 (58%)
Missing answers	0 (0%)	1 (17%)	0 (0%)	0 (0%)	1 (4%)
Health literacy					
Low: n (%)	0 (0%)	1 (17%)	1 (17%)	1 (17%)	3 (13%)
Moderate: n (%)	4 (67%)	2 (33%)	2 (33%)	2 (33%)	10 (42%)
High: n (%)	2 (33%)	3 (50%)	3 (50%)	3 (50%)	11 (46%)

Appendix 10: Participants' grading of treatment characteristics in order of importance from highest to lowest importance

Participants were asked to grade the characteristics as a preparation for the discussion according to how important they found them, on a scale from 1 – 5 (1= not important at all; 2 = not important; 3 = neutral; 4 = important; 5 = very important).

Characteristic in order of importance, based on average grade	Average	Median	Standard deviation	Highest grade	Lowest grade	Difference highest – lowest grade
Progression free survival	4,87	5,00	0,34	5,00	4,00	1,00
Cancer	4,75	5,00	0,53	5,00	3,00	2,00
Stroke	4,74	5,00	0,54	5,00	3,00	2,00
Response to treatment	4,71	5,00	0,62	5,00	3,00	2,00
Overall survival	4,70	5,00	0,63	5,00	3,00	2,00
Bone damage and bone fractures	4,42	5,00	0,93	5,00	2,00	3,00
Quality of life	4,42	4,00	0,58	5,00	3,00	2,00
Confusion	4,33	4,00	0,70	5,00	3,00	2,00
Low level of consciousness	4,31	4,00	0,62	5,00	3,00	2,00
Memory loss or amnesia	4,25	4,00	0,85	5,00	2,00	3,00
Hallucinations	4,17	4,00	0,82	5,00	3,00	2,00

Thinking disorders	4,17	4,00	0,87	5,00	2,00	3,00
Heart failure	4,13	4,50	0,99	5,00	2,00	3,00
Sepsis	4,13	4,00	1,03	5,00	1,00	4,00
Infections	4,04	4,00	0,91	5,00	2,00	3,00
Bone or back pain	3,98	4,00	0,70	5,00	3,00	2,00
Fatigue	3,98	4,00	0,81	5,00	2,00	3,00
Change in psychological and mental state	3,92	4,00	0,83	5,00	2,00	3,00
Neuropathic pain	3,77	4,00	0,63	5,00	2,00	3,00
Tremor	3,71	4,00	0,81	5,00	2,00	3,00
Dizziness	3,63	4,00	0,88	5,00	2,00	3,00
Cough, wheezing and dyspnea	3,54	4,00	1,06	5,00	1,00	4,00
Fever	3,33	3,00	0,92	5,00	2,00	3,00
Insomnia	3,29	3,00	0,95	5,00	1,00	4,00
Diarrea	3,29	3,00	0,95	5,00	1,00	4,00
Hypertension or hypotension	3,25	3,00	0,99	5,00	1,00	4,00
Skin numbness	3,23	3,00	0,72	5,00	2,00	3,00
Aritmia	3,23	3,00	0,93	5,00	1,00	4,00

Edema	3,21	3,00	0,83	5,00	1,00	4,00
Muscle weakness or muscle cramps / spasms	3,19	3,00	0,76	5,00	2,00	3,00
Chest pain	3,17	3,00	0,87	4,00	1,00	3,00
Nausea	3,17	3,00	1,17	5,00	1,00	4,00
Bleeding	3,17	3,00	1,09	5,00	1,00	4,00
Constipation	3,04	3,00	0,98	5,00	1,00	4,00
Weight loss	3,04	3,00	0,91	4,00	1,00	3,00
Tingling	3,04	3,00	0,95	5,00	1,00	4,00
Decreased appetite	2,96	3,00	1,00	5,00	1,00	4,00
Headache	2,83	3,00	0,96	5,00	1,00	4,00
Rash	2,63	2,00	1,06	5,00	1,00	4,00

References

1. Velcade (bortezomib) dosing, indications, interactions, adverse effects, and more: http://reference.medscape.com; 2019 [Available from: https://reference.medscape.com/drug/velcade-bortezomib-342256#4.
2. Revlimid® Treatment and Multiple Myeloma 2019 [Available from: https://news.cancerconnect.com/multiple-myeloma/revlimid-treatment-and-multiple-myeloma-xYSTxwNjRkiqSZ7GwmrkMQ/.
3. Revlimid (lenalidomide) dosing, indications, interactions, adverse effects, and more. 2019.
4. Kyprolis (carfilzomib) dosing, indications, interactions, adverse effects, and more: http://reference.medscape.com; 2019 [Available from: https://reference.medscape.com/drug/kyprolis-carfilzomib-999762#4.
5. Darzalex (daratumumab) dosing, indications, interactions, adverse effects, and more. 2019.
6. Xiao Y, Yin J, Wei J, Shang Z. Incidence and risk of cardiotoxicity associated with bortezomib in the treatment of cancer: a systematic review and meta-analysis. PloS one. 2014;9(1):e87671-e.
7. Ludwig H, Delforge M, Facon T, Einsele H, Gay F, Moreau P, et al. Prevention and management of adverse events of novel agents in multiple myeloma: a consensus of the European Myeloma Network. Leukemia. 2018;32(7):1542-60.
8. Spotten LE, School of Biological Sciences DIoTD, Faculty of Health Sciences TCDD, Corish CA, School of Public Health P, Sports Science UCDD, et al. Subjective and objective taste and smell changes in cancer. Annals of Oncology. 2019;28(5):969-84.
9. Ramsenthaler C, Osborne TR, Gao W, Siegert RJ, Edmonds PM, Schey SA, et al. The impact of disease-related symptoms and palliative care concerns on health-related quality of life in multiple myeloma: a multi-centre study. BMC cancer. 2016;16:427-.
10. Raje N, Berdeja J, Lin Y, Siegel D, Jagannath S, Madduri D, et al. Anti-BCMA CAR T-Cell Therapy bb2121 in Relapsed or Refractory Multiple Myeloma. The New England journal of medicine. 2019;380(18):1726-37.
11. Ramsenthaler C, Osborne TR, Gao W, Siegert RJ, Edmonds PM, Schey SA, et al. The impact of disease-related symptoms and palliative care concerns on health-related quality of life in multiple myeloma: a multi-centre study. BMC cancer. 2016;16:427-.
